# Different chemical scaffolds bind to L-phe site in *Mycobacterium tuberculosis* Phe-tRNA synthetase

**DOI:** 10.1016/j.ejmech.2025.117335

**Published:** 2025-01-31

**Authors:** Priyanka Gade, Changsoo Chang, Denise S. Pryde, Daniel Fletcher, Sarah Niven, Luma Godoy Magalhaes, David Robinson, Jagmohan Saini, Peter E.G.F. Ibrahim, Barbara Forte, Jacek Wower, Michael J. Bodkin, Beatriz Baragaña, Ian H. Gilbert, Karolina Michalska, Andrzej Joachimiak

**Affiliations:** a Center for Structural Biology of Infectious Diseases, Consortium for Advanced Science and Engineering, University of Chicago, Chicago, IL, 60667, USA; b Structural Biology Center, X-ray Science Division, Argonne National Laboratory, Lemont, IL, 60439, USA; c Drug Discovery Unit, Wellcome Centre for Anti-Infectives Research, Division of Biological Chemistry and Drug Discovery, University of Dundee, Dundee, DD1 5EH, UK; d Department of Animal Sciences, Auburn University, Auburn, AL, 36849, USA; e Department of Biochemistry and Molecular Biology, University of Chicago, Chicago, IL, 60367, USA

## Abstract

Tuberculosis (TB), caused by *Mycobacterium tuberculosis* (*Mt*), is one of the deadliest infectious diseases. The rise of multidrug-resistant strains represents a major public health threat, requiring new therapeutic options. Bacterial aminoacyl-tRNA synthetases (aaRS) have been shown to be highly promising drug targets, including for TB treatment. These enzymes play an essential role in translating the DNA gene code into protein sequence by attaching specific amino acid to their cognate tRNAs. They have multiple binding sites that can be targeted for inhibitor discovery: amino acid binding pocket, ATP binding pocket, tRNA binding site and an editing domain. Recently we reported several high-resolution structures of *M. tuberculosis* phenylalanyl-tRNA synthetase (*Mt*PheRS) complexed with tRNA^Phe^ and either L-Phe or a nonhydrolyzable phenylalanine adenylate analog. Here, using Nucleic Magnetic Resonance (NMR) and Surface Plasmon Resonance (SPR) we identified fragments that bind to *Mt*PheRS and we determined crystal structures of their complexes with *Mt*PheRS/tRNA^Phe^. All the binders interact with the L-Phe amino acid binding site. The analysis of interactions of the new compounds combined with adenylate analog structure provides insights for the rational design of anti-tuberculosis drugs. The 3′ arm of the tRNA^Phe^ in all the structures was disordered with exception of one complex with D-735 compound. In this structure the 3’ CCA end of the acceptor stem is observed in the editing domain of *Mt*PheRS providing insights regarding the post-transfer editing activity of class II aaRS.

## 1. Introduction

Tuberculosis (TB), caused by *Mycobacterium tuberculosis* (*Mt*), is the second, after COVID-19, most lethal disease resulting in 1.25 million deaths in 2023 [1]. The COVID-19 pandemic has worsened the occurrence and mortality of TB, as the interrupted routine screening programs led to reduced detection [2,3]. This alarming situation amplifies an already urgent need for novel anti-TB drugs, which will increase efficacy, reduce the treatment time and cost and combat the rising clinical drug resistance. Targeting protein translation, an essential process in all cellular organisms, is an important direction in developing antibacterial treatments. Linezolid, an FDA-approved antibiotic against Gram-positive bacteria, including *Mt*, binds to a site on the 23S rRNA of the 50S ribosomal subunit and blocks the formation of a functional 70S initiation complex. Several other anti-TB compounds presently tested in clinical trials also target protein translation [4,5]. Aminoacyl-tRNA synthetases (aaRSs), which are indispensable for protein synthesis, are attractive targets for the identification of novel anti-bacterial drugs [6, 7]. The evolutionary divergence between prokaryotic and eukaryotic aaRSs enables the development of selective inhibitors of bacterial aaRSs [8]. At the same time, high structural conservation of aaRSs across prokaryotes can lead to broad-spectrum antibacterials. Recently, the development of a drug-like series of inhibitors of *Mt*lysyl-tRNA synthetase (LysRS) with efficacy in mouse models of infection has been reported [9]. At a more advanced stage of drug development, GSK3036656, a compound inhibiting *Mt*leucyl-tRNA synthetase, has emerged as a promising anti-TB candidate and is currently undergoing clinical trials [10] (Supplementary Fig. S1).

AaRSs catalyze the attachment of the correct amino acid to its cognate tRNA which then is used in protein synthesis on ribosomes. The aminoacylation of tRNA by aaRS is carried out in two steps. First, the specific amino acid residue is activated by forming an aminoacyl-AMP adduct using ATP and releasing pyrophosphate. The tRNA is then aminoacylated by esterification at the ribose of 3′-end adenosine of the acceptor stem of the cognate tRNA [11]. Based on the evolution and active site topology, bacterial aaRSs enzymes are classified into two main classes, class I and class II [12]. These two classes of aaRS approach the acceptor stem of tRNA differently and as result class I enzymes attach amino acid to the 2′-OH and class II to 3′-OH moieties of the tRNA’s 3′-terminal ribose, respectively. Phylogenetically phenylalanyl-tRNA synthetase (PheRS), belongs to the class II aaRSs, and it is a dimer of heterodimers ${\left(\text{αβ}\right)}_{2}$, containing two α- (PheS) and two β- (PheT) subunits [13]. Structural studies of PheRS have shown that the α subunit binds L-Phe and ATP and is responsible for esterification catalysis. At the same time, the β subunit is involved in the recognition of the cognate tRNA molecule and editing of mis-charged tRNA [13–17]. Interestingly, PheRS is the sole member of class II enzymes that attaches the amino acid to the 2′-OH group, rather than 3′-OH, of the tRNA’s 3′-terminal ribose [18]. Among the 20 bacterial aaRS, PheRS offers unique features. Its large, heterotetrametric structure is shared only with glycyl-tRNA synthetase (GlyRS) [19–22]. Even though the structural architecture of the bacterial PheRS is similar to the eukaryotic cytosolic PheRS, functionally important active site residues are found to be divergent [23]. Also, the human mitochondrial PheRS has evolved to a simplified monomeric chimera comprising of the catalytic module of the α-subunit and the C-terminal tRNA binding domain of the bacterial β-subunit [24, 25]. Similarity of the adenylate binding cavity between *Mt*PheRS and mitochondrial homolog represents a daunting challenge for medicinal chemistry, fortunately mitochondrial enzyme is missing the editing domain.

PheRS stands out in various high-throughput drug screening assays as a promising antibacterial target [26–28]. Among various PheRS inhibitors discovered so far, phenyl–thiazolylurea–sulfonamides provided high potency and broad-spectrum antibacterial activity [28,29]. However, these compounds were not anti-TB specific, except for GDI05–001 [30]. Isopatulin, a natural product isolated from the fungal strain *Penicillium griseofulvum* CPCC-400528 inhibited PheRS from *Mt*H37Rv strain [31]. Also, the compound PF-3845 was identified in a screen of a bioactive compound library, through non-nucleoside bisubstrate competitive binding assay [30].

Since editing function in only occasionally executed, targeting the enzyme’s essential functions offers more reliable strategy for an antibacterial drug. Thus, this study aims to identify chemical scaffolds that can bind to L-Phe and ATP binding sites and potentially inhibit *Mt*PheRS. To achieve this, we utilized Phe-AMS, a known inhibitor that binds to the active site, and performed fragment screening using NMR. The fragments that can competitively displace Phe-AMS are considered active-site binding fragments and can serve as the basis for developing more potent inhibitors. Using NMR, we screened the DDU fragment library (1072 fragments), and positive hits were followed by Surface Plasmon Resonance (SPR) studies to prioritize a subset of compounds for crystallography. The protein-fragment interactions were analyzed using crystal structures for five of those fragments complexed with *Mt*PheRS/tRNA^Phe^ (DDD00107555 (D-555), DDD01008876 (D-876), DDD00805735 (D-735), DDD00079004 (D-004) and DDD00067116 (D-116)). The fragments were further evaluated for their inhibitory activity for the activation of L-Phe. While these five fragments bind to the phenylalanine binding pocket, their distinct scaffolds offer opportunities for the development of new potent inhibitors of *Mt*PheRS as the next-generation anti-TB drugs.

Although the active site pockets of aaRSs are finely tuned to preferentially bind the correct amino acid and effectively reject non-cognate compounds, this discrimination is not perfect, and mistranslation can impact the accurate flow of information between gene and protein. To correct mis-acylation and ensure the high fidelity of translation, aaRSs have evolved proofreading mechanisms that fall into two primary categories: pre-transfer editing and post-transfer editing. Pre-transfer editing mechanisms operate within the acylation site of the aaRSs, performing hydrolysis of misactivated aminoacyl adenylates before they are transferred onto tRNA. In contrast, post-transfer editing mechanisms involve dedicated domains within the aaRSs designed to remove incorrect amino acids that have been wrongly attached to tRNA. Post-transfer editing activity of PheRS has been described, in which the transfer of tyrosine to tRNA^Phe^ was demonstrated and the misacylated tRNA is rapidly hydrolyzed [32]. Later, this editing activity of the bacterial and archaeal/eukaryotic PheRSs was associated with the specific site in the β subunit [16,33]. In the present study, the 3’ CCA tail of the tRNA^Phe^ in the αβ heterodimer bound to D-735 was translocated into the editing domain, revealing the tRNA conformation during post-transfer editing. Our work provides the mechanistic insights of the previously proposed shuttle proofreading mechanism for class II aaRS.

## 2. Experimental procedures

### 2.1. Expression and purification of MtPheRS

The expression plasmids described in the previous study [17] were used for protein production in *Escherichia coli* BL21-Gold (DE3). Briefly, *pheS* gene encoding the α-subunit was cloned to the pMCSG53 vector, while the *pheT* encoding the β subunit was cloned into pMCSG120. Vectors pMCSG53 and pMCSG120 carry ampicillin and kanamycin resistance respectively. In the final construct, the *pheS* sequence is amended on the N-terminus with the His_6_ affinity tag followed by Tobacco Etch Virus (TEV) protease cleavage site. The *Mt*PheRS was overexpressed and purified according to the previously described protocol [17]. *E. coli* BL21-Gold (DE3) competent cell was co-transformed with the PheS-pMCSG53 and PheT-pMCSG120 expression plasmids and was grown at 37 °C overnight in LB medium containing 150 μg/ml ampicillin and 100 μg/ml kanamycin. 10 ml of the overnight culture was inoculated to 1liter LB medium containing antibiotics and grown at 37 °C until the OD_600_ reached 0.8–1.0. The culture was cooled down to 18 °C, followed by the addition of IPTG to the final concentration of 0.25 mM. The culture was further grown at 18 °C for 20 h, harvested by centrifugation (3700×*g* for 20 min at 4 °C) and resuspended with buffer A containing 20 mM HEPES pH 7.5, 500 mM NaCl, 20 mM imidazole at pH 8.0, 5 % glycerol and 10 mM 2-mercaptoethanol. Cells were disrupted on ice using sonication (5 min total time, 130 W power output, 60 % amplitude), followed by centrifugation at 30,000×*g* for 80 min at 4 °C.

The protein was purified by Ni^2+^ immobilized metal affinity chromatography (IMAC) using 5 ml Ni^2+^ Sepharose (GE Healthcare Life Sciences) in a 2.5-cm × 10-cm Flex-Column (420400–2510) connected to a Vac-Man vacuum manifold (Promega). The supernatant was loaded onto the column (GE Healthcare Life Sciences) equilibrated with buffer A and mixed thoroughly with the resin. The supernatant was removed using vacuum of 15 p.s.i. Unbound proteins were washed out with 100 ml buffer A and then buffer B (20 mM HEPES pH 7.5, 500 mM NaCl, 50 mM imidazole at pH 8.0, 5 % glycerol and 10 mM 2-mercaptoethanol). The *Mt*PheRS ${\left(\text{αβ}\right)}_{2}$ complex was eluted using 15 ml buffer A supplemented with 500 mM imidazole at pH 8.0. The N-terminal His-tag of the PheS was cleaved overnight by TEV protease at a molar ratio of 1:20. The FT was concentrated to about 2 ml and loaded onto Superdex 200 16/70 size exclusion column (GE Healthcare Life Sciences) equilibrated with crystallization buffer containing 20 mM HEPES at pH 7.5, 250 mM KCl, 1 mM MgCl_2_ and 1 mM TCEP. Fractions containing *Mt*PheRS heteroteramer were collected and concentrated to ~30 mg/ml using Amicon 30-kDa-cutoff concentrators (Millipore).

### 2.2. Ligand observed NMR fragment screen

A screen of the DDU fragment library (1072 fragments) was conducted on a Bruker 500 MHz (AVANCE III) with Cryo platform using STD, WaterLOGSY and CPMG experiments conducted with and without the presence of an inhibitor. Samples contained 5 μM *Mt*PheRS protein in 50 mM Tris, 150 mM NaCl and 1 mM TCEP buffer with 0.5 mM ligand in pools of 6–8 fragments. Following the acquisition of initial data set, 40 μM 5′-O-[N-(phenylalanine) sulfamoyl] adenosine (Phe-AMS) was added in a competition experiment. Data were analyzed using Topspin 3.6.1. to identify which fragments demonstrated binding and competition with more confidence given to those which show evidence of binding in all experiments and displacement with addition of Phe-AMS. Data sets were compared with proton reference spectra of each fragment in the pool of fragments in the sample to identify the data pertaining to each fragment within the pool.

### 2.3. Surface Plasmon Resonance (SPR) binding assay

In a Biacore^™^ T200 (Equipment No. 16368) SPR instrument, *Mt*PheRS protein (0.08 mg/ml) was immobilized onto FC2 of a Series S NTA chip (GE Healthcare Life Sciences) by nickel and amine coupling (GE Healthcare Life Sciences) until a response of 7970 RU was obtained for experiment number one and 6146 RU was obtained for experiment number two. FC1 was used as reference surface. The buffer containing 100 mM HEPES at pH 7.4, 10 mM NaCl, 5 mM MgCl_2_, 50 μM EDTA and 0.05 % P20 was used for immobilization. A concentration series was prepared for the compounds as a 5-point, 3-fold dilution (top concentration 1 mM) in immobilization buffer plus DMSO. DMSO concentration was kept constant at 3 % in all samples. Blank buffer solutions were run between each compound for double subtraction (FC 2–1 = first subtraction, compound cycle-blank cycle = second subtraction). The running buffer used in the assay consists of 100 mM HEPES at pH 7.4, 10 mM NaCl, 5 mM MgCl_2_, 50 μM EDTA, 0.05 % P20 and 3 % DMSO. Samples were injected at 30 μl/min over the *Mt*PheRS surface and the reference surface with an association phase of 30 s followed by a dissociation phase of 30 s for each compound concentration. An 8-point DMSO calibration curve was also included (solvent correction, DMSO 2.5–3.8 %). A control compound, Phe-AMS, was injected at 10 nM concentration every 40 cycles. Phe-AMS consistently showed binding at expected levels during the whole experimental run, indicating adequate protein stability on the chip.

Data was analyzed using Biacore^™^ Insight Evaluation Software 5.0.18.22102. The cut-off for compound response was established using the binding level screen boundary tool implemented on the software.

### 2.4. Crystallization of MtPheRS/tRNA^Phe^

The *Mt*tRNA^Phe^ used for crystallization studies was synthesized using the previously described method [17]. Purified *Mt*PheRS at 15 mg/ml concentration was mixed with tRNA^Phe^ in 1:2 M ratio (protein to tRNA^Phe^). Crystallization experiments were conducted using 96-well CrystalQuick plates (Greiner Bio-One), where 0.3 μl *Mt*PheRS/tRNA^Phe^ solution and 0.3 μl crystallization solution were mixed and equilibrated against 120 μl of corresponding well solution at 16 °C. Sitting drop vapor diffusion experiments were carried out using a Mosquito liquid dispenser (TTP LabTech) and sparse matrix screens (Qiagen). Rod-shaped crystals were obtained in 0.2 M ammonium acetate, 0.1 M HEPES pH 7.5 and 25 % w/v PEG 3350 condition. For soaking experiments, crystals were transferred into the drops consisting of 1 μl reservoir solution supplemented with 10 mM compound. Crystals were harvested after 2 min of soaking, cryoprotected using reservoir solution supplemented with 15 % v/v ethylene glycol, and flash cooled. A stock solution of 100 mM fragments was prepared using DMSO solvent.

### 2.5. Data collection, refinement, and structure analysis

Diffraction data were collected at the 19-ID beamline of the Structural Biology Center (SBC) at the Advanced Photon Source, Argonne National Laboratory. Data collection was conducted at 100K, and the diffraction images were recorded using a PILATUS3 X 6 M detector. Diffraction intensities were evaluated and integrated using the HKL3000 suite [34]. Intensities were converted to structure factor amplitudes in the Ctruncate program [35,36] from the CCP4 package [37]. These crystals belong to the monoclinic P2_1_ space group and contain entire *Mt*PheRS ${\left(\text{αβ}\right)}_{2}$/(tRNA^Phe^)_2_ complex in the asymmetric unit. The *Mt*PheRS/tRNA^Phe^ structure was determined by molecular replacement with Phaser [38] using *Mt*PheRS/tRNA^Phe^ (PDB ID: 7KA0) as a search model. Manual model building was performed using COOT [39] and the structure was refined using Phenix [40]. Ligand dictionaries were prepared using AceDRG [41] or eLBOW module [42] of Phenix and model quality was assessed using Molprobity [43]. The data collection and refinement statistics are summarized in Table 1. Structural analysis was conducted and figures were prepared using PLIP [44] and PyMol software [45].

### 2.6. Binding energy calculation

To evaluate the binding energies between the fragments and the residues in the binding site of *Mt*PheRS, we performed Quantum Mechanics Fragment Molecular Orbital (QM-FMO) using the fragment bound structures of *Mt*PheRS/tRNA^Phe^. QM-FMO is used for calculating the binding interaction energies described by the Inter-Fragment Interaction Energy (IFIE) and its Pair Interaction Energy Decomposition Analysis (PIEDA) [46,47]. The IFIE analysis is a computational method used to estimate protein–ligand interactions, focusing on the contribution of quantum (electronic) effects. This analysis provides valuable insights into molecular interactions while maintaining a moderate level of computational complexity. One of the key components of IFIE analysis is PIEDA, which allows for the decomposition of the total FMO interaction energy $\left(\Delta E^{FMO}\right)$ into several contributing energy terms including electrostatic $\left(\Delta E_{IJ}^{ES}\right)$, exchange repulsion $\left(\Delta E_{IJ}^{EX}\right)$, dispersion $\left(\Delta E_{IJ}^{DI}\right)$, charge transfer with higher-order mixed terms $\left(\Delta E_{IJ}^{CT+mix}\right)$, and solvation energy $\left(\Delta E_{IJ}^{Gsol}\right)$, as shown in equation (1). The $\Delta E^{FMO}$ is calculated as the sum of these energy terms, providing a comprehensive understanding of the protein-ligand interactions. Notably, this analysis is performed in an implicit water environment, utilizing a polarizable continuum model (PCM), and has been instrumental in various drug discovery projects, offering detailed insights into molecular interactions and aiding in the design of novel therapeutics.

Equation (1) $$\text{Δ}E^{FMO}=\text{Δ}E_{IJ}^{ES}+\text{Δ}E_{IJ}^{EX}+\text{Δ}E_{IJ}^{CT+mix}+\text{Δ}E_{IJ}^{DI}+\text{Δ}E^{Gsol}$$

$\text{Δ}E^{FMO}$: Interaction energy of calculation.

Protein residues within a radius of 5 Å from the ligand atoms were included in the $\text{Δ}E^{FMO}$ calculations. The C-terminal carboxylic acid of the peptide was capped with N-methylamine, and the N-terminal position acetylated while maintaining the geometry of the neighboring residues. A given amino acid, along with its side chain, C-alpha, backbone NH, and the carbonyl group of the adjacent amino acid, define each FMO fragment. Fragmentation was carried out according to a well-established fragmentation strategy inspired from Facio [48], in a fully automated high-throughput python code, each residue (fragment) was defined as an amino acid, its side chain, C-alpha, the backbone NH, and the carbonyl group of the subsequent amino acid. The calculations were performed at second order density function tight-binding (FMO2-DFTB) theory level, using General Atomic and Molecular Electronic Structure System (GAMESS) implementation [49,50].

### 2.7. FMO calculations

The fragment bound $Mt{\text{PheRS/tRNA}}^{\text{Phe}}$ structures were initially processed using the Protein Preparation module in the Schrödinger suite using the default parameters. During this step, the water molecules within 5 Å of the co-crystallized fragment is not retained as part of the structure and their hydrogen bond network is optimized. The complexes thus generated were minimized with the OPLS3e force field using MacroModel from the Schrödinger suite of programs. From the prepared complexes, the atoms of the residues within 5 Å from the fragment were selected for the FMO. However, to improve the speed while performing QM calculations these were removed from individual complexes.

The FMO calculations were performed using GAMESS [51,52]. As a default, we used the two-body implementation of the FMO method with the Density Functional Based Tight Binding (DFTB) method approximates density functional theory (DFT) as the quantum mechanical level of theory [53]. This choice was used to combine accuracy and computational cost within the framework of the FMO method. All calculations were performed on a hybrid CPU/GPU cluster equipped with 16 GPUs (2 NVIDIA GTX 1080 per node on 8 GPUs).

Furthermore, ligand efficiency was calculated for individual fragments using formula (equation (2))

Equation (2) $$\frac{\text{Δ}E^{FMO}}{HAC}$$

$\text{Δ}E^{FMO}$: The interaction energy of a ligand, HAC: Heavy atom count.

### 2.8. Aminoacylation inhibition assay

Aminoacylation inhibition was monitored by a modified assay that has been described by Bullock et al. [54]. Reaction mixtures consisted of 50 mM HEPES/KOH pH 7.5, 25 mM KCl, 10 mM MgCl_2_, 5 mM DTT, 100 μM L-Phe, 20 μM *Mt*tRNA^Phe^ transcript, 1.5 μM *Mt*PheRS, 3- and 4.5-mM fragment compounds and 5 % and 10 % (v/v) DMSO, respectively. Reactions were first incubated for 10 min on ice to allow for the binding of fragments and initiated with the addition of 1.5 mM ATP/0.25 μM [α−32P] ATP. Then, reactions were incubated for 30 min at 37 °C. All reactions were quenched in 400 mM sodium acetate (pH 5.2) with 0.1 % (w/v) SDS. Aliquots were spotted on polyethyleneimine-cellulose TLC plates (Macherey and Nagel), developed in 100 mM ammonium acetate, 5 % (v/v) acetic acid and analyzed in a scintillation counter.

## 3. Results

### 3.1. Identification of novel MtPheRS binding fragments by NMR and validation by SPR

**Fig. 1.**
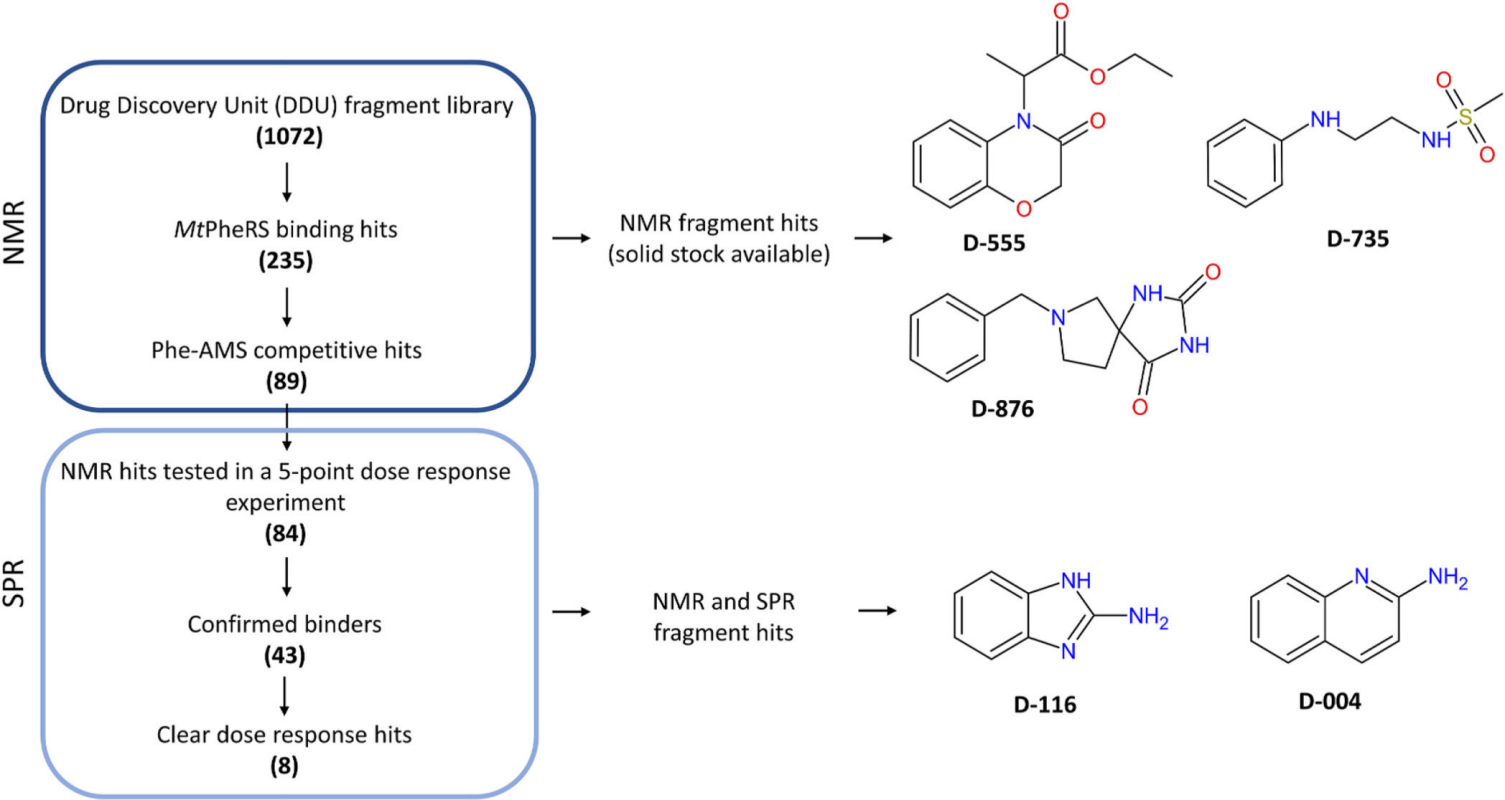
Schematic summary of fragment screening approach of *Mt*PheRS using NMR and SPR. Total of 28 fragment hits binding *Mt*PheRS were selected for X-ray crystallography studies. Chemical structures of fragments are shown.

A ligand observed NMR screen of the DDU fragment library [55] (1072 fragments selected based on physicochemical properties from commercial sources or prepared in house) identified 235 fragments displaying binding to *Mt*PheRS in all three NMR runs. Out of these 235 fragments, 89 hits were displaced upon addition of Phe-AMS, suggestive of specific active site binding (Fig. 1, Supplementary Fig. S2 and Supplementary Table S1). The 84 out of the 89 fragments that demonstrated competitive binding with Phe-AMS in the NMR screen were further evaluated by SPR. Two SPR experiments were performed independently. Across both runs, 43 fragments consistently showed response above cut-off (established as described in the method section) for the highest concentration of compound (1 mM) and 10 fragments presented response above cut-off for at least the two highest compound concentrations (1 and 0.33 mM) (Supplementary Fig. S3). All responses were below the theoretical R_max_ value for each fragment suggestive of specific binding. R_max_ indicates the maximum response expected for a ligand based on the protein and ligand molecular weights and level of protein capture to the chip. R_max_ greater than the theoretical value is indicative of non-specific binding or aggregation.

Although K_D_ values could not be confidently determined using either a kinetic fit or steady state analysis due to the weak binding nature of fragments, eight fragments showed dose-response binding for at least two concentrations in both SPR experiments. Among those 8 fragments, DDD01008820 and DDD01305616 showed slower off rates (Supplementary Fig. S4).

We selected 22 NMR fragments hits that were available in house as solids for crystallization trials, and of those, two had showed dose-response binding in SPR experiments. In addition, we purchased the remaining six fragments that showed dose-response binding by SPR to progress to crystallization trials too.

### 3.2. Crystallization of MtPheRS/tRNA^Phe^ inhibitor complexes

The α- (PheS) and β- (PheT) subunits of *Mt*PheRS were co-expressed from individual vectors in *E. coli* and recombinant proteins assembled into functional heterotetramer ${\left(\text{αβ}\right)}_{2}$. The protein was purified as described previously [17] and the size of complex was confirmed by the size exclusion chromatography. To facilitate a structure-guided approach for the development of new antibacterial compounds, we first crystallized the apo form of *Mt*PheRS bound to tRNA^Phe^. Crystals of *Mt*PheRS/tRNA^Phe^ complex were used to perform soaking experiments with chemical fragments. These crystals were exposed to X-ray beam at SBC 19-ID beamline and data were collected for the crystals diffracting beyond 3.0 Å resolution.

### 3.3. Overall structure of MtPheRS/tRNA^Phe^ inhibitor complex

**Fig. 2.**
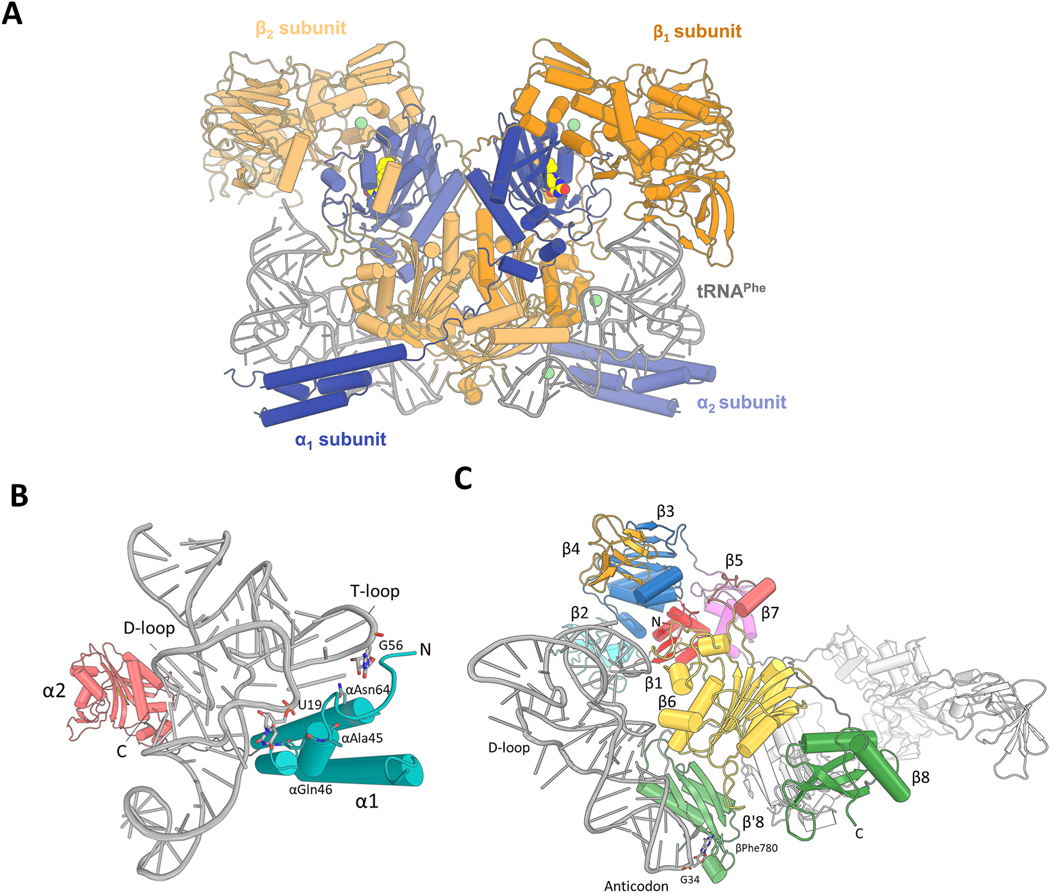
Structure of *Mt*PheRS/tRNA^Phe^ complex bound to D-876 fragment. (**A**) Ribbon representation of the crystal structure of the heterotetrameric *Mt*PheRS/tRNA^Phe^ complex bound to D-876 fragment. Blue, α subunit; orange, β subunit; gray, tRNA^Phe^. Mg^2+^ ions are represented as light green spheres. The fragment D-876 is shown as spheres. (**B**) α subunit of *Mt*PheRS. Cyan, α1 domain; salmon, α2 domain. Linker region connecting α1 and α2 domains is colored in dark gray. Protein residues and the tRNA^Phe^ interacting nucleotides are shown as sticks. (**C**) β subunit of *Mt*PheRS. Red, β1; cyan, β2; blue, β3; orange, β4; magenta, β5; yellow, β6; salmon, β7; green, β8 and ${\text{β}}^{\prime }8$. The conserved βPhe780 residue interacting with the anticodon G34 nucleotide of tRNA^Phe^ is shown in sticks. (For interpretation of the references to color in this figure legend, the reader is referred to the Web version of this article.)

We obtained the crystal structures of *Mt*PheRS/tRNA^Phe^ complex bound to five different fragments (Fig. 1). The crystals belong to the space group P2_1_ with one heterotetrameric molecule of *Mt*PheRS bound with two *Mt*tRNA^Phe^ in the asymmetric unit. The structures were solved by molecular replacement (MR) using *Mt*PheRS/tRNA^Phe^ complex (PDB ID: 7KA0) as a search model. The final refined models have reasonable R-factors and good geometry (Table 1). Difference Fourier maps (mF_o_-DF_c_) with amplitudes and phases based on the refined *Mt*PheRS/tRNA^Phe^ model showed the presence of strong extra positive electron density corresponding to soaked fragment molecules. Crystallographic data collection and refinement statistics are shown in Table 1. Similar to previous studies, the *Mt*PheRS is a functional heterodimer, that assembles into ${\left(\text{αβ}\right)}_{2}$ heterotetramer (Fig. 2A and Supplementary Fig. S5). The overall binding of the tRNA^Phe^ to PheRS is similar to previously reported structures of PheRS/tRNA^Phe^ complexes with either L-Phe or Phe-AMP or PheOH-AMP, or Phe-AMS [13,15,17,56,57]. Both *Mt*tRNA^Phe^ molecules interact extensively with all the four subunits of the heterotetramer in all the five structures. The electron density of the residues in both the α and β subunits is excellent in all the five structures except for a few residues that were not modeled (Supplementary Table S2). The smaller α subunit is comprised of two domains; α1 and α2, which are connected by a flexible linker (residues 94–107) (Fig. 2B). In all structures, the binding of tRNA^Phe^ to PheRS is virtually identical (with one exception, see results below for D-735 fragment), indicating that binding small fragments to the L-Phe binding site does not impact the enzyme/tRNA^Phe^ interaction. The N-terminal helical arm of the α1 domain interacts with the D- and T-loop regions of the tRNA^Phe^, while the residues in the C-terminal of the α2 domain are part of the aminoacylation catalytic pocket (discussed below). The αAla45 and αGln46 residues make hydrogen bond interactions with the U19 base of the D-loop in all five structures. The αAsn64 stabilize the T-loop of tRNA^Phe^ through hydrogen bond interactions (Fig. 2B). The larger globular β subunit containing eight domains (β1-β8) recognizes and binds tRNA^Phe^ and performs proof reading (Fig. 2C). The conserved βPhe780 residue in the β8 anticodon binding domain makes stacking interactions with the G34 anticodon base of tRNA^Phe^. An extensive network of hydrogen bond interaction between the residues of the β՛8 domain and the anticodon region of tRNA^Phe^ is observed. Furthermore, the D-loop is stabilized by hydrogen bond interaction with the residues from the β6 and β8 domains. This detailed network of interactions between *Mt*PheRS and tRNA^Phe^ is shown in Supplementary Fig. S6. The electron density of the 3’ end of the acceptor stem of tRNA^Phe^ is not visible (with the exception of one αβ heterodimer *Mt*PheRS/tRNA^Phe^ complex bound to D-735) and therefore is not modeled.

The *Mt*PheRS/tRNA^Phe^ heterotetrametric architecture of all five structures superimpose well with the previously determined structures of *Mt*PheRS/tRNA^Phe^ bound to L-Phe (PDB ID: 7KA0, average rmsd – 1.15 Å over 1944 ${\text{C}}_{\text{α}}$ atoms) and nonhydrolyzable phenylalanine adenylate analog (Phe-AMS) (PDB ID: 7K98; average rmsd – 0.81 Å over 2073 ${\text{C}}_{\text{α}}$ atoms), respectively (Supplementary Fig. S7).

### 3.4. Fragment binding and FMO calculations

At the given resolution, we were able to observe the electron density corresponding to D-555, D-876, D-735, D-116 and D-004 fragments in both the ${\text{α}}_{1}$ and ${\text{α}}_{2}$ subunits of *Mt*PheRS (Supplementary Fig. S8). These five fragments represent different chemical scaffolds. All five fragments occupy the L-Phe site in the α subunit of the heterotetramer. The fragments’ orientation in these structures is very similar to the L-Phe substrate, however there are subtle differences in orientation and details of interactions (Supplementary Fig. S9).

**Fig. 3.**
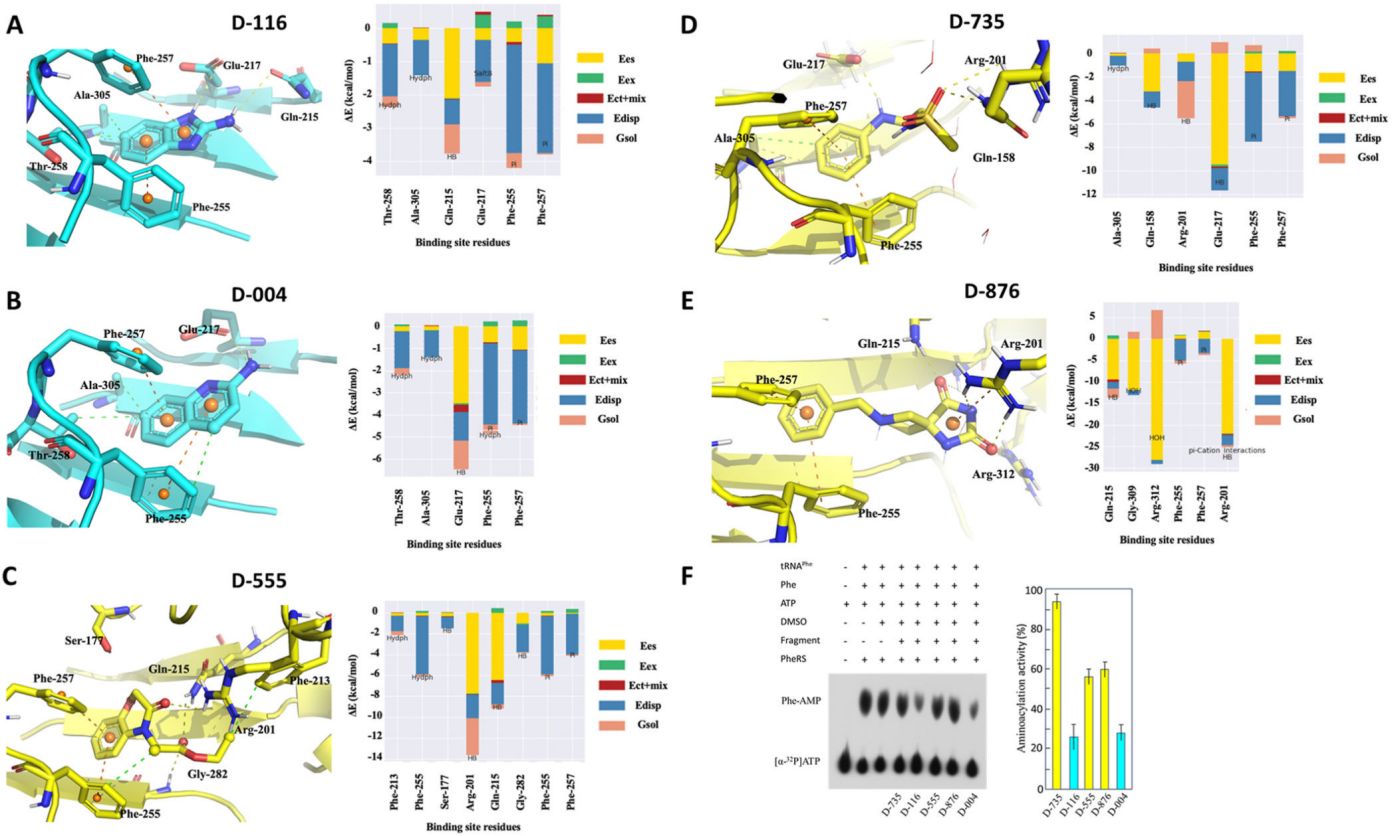
Close-up view of (**A**) D-116, (**B**) D-004, (**C**) D-555, (**D**) D-735, (**E**) D-876 fragments interaction around the L-Phe binding pocket of *Mt*PheRS interpreted from FMO analysis along with the PEIDA interaction energies. Fragments determined by NMR and SPR are shown as yellow and cyan respectively. (**F**) Aminoacylation inhibition by 3.0 mM fragments in the presence of 5 % DMSO. *Mt*PheRS retained 94, 26, 56, 60, and 28 % aminoacylation activity in presence of D-735, D-116, D-555, D-876, and D-004 fragments respectively. (For interpretation of the references to color in this figure legend, the reader is referred to the Web version of this article.)

An extensive network of interactions is observed between the fragments and the active site residues in the α2 domain of the α subunit. Using FMO analysis, these fragment/PheRS interactions were described in terms of PIEDA, where all five fragments were plotted separately to identify the energy components of interaction. Hydrogen bond interaction and salt bridges refer to the electrostatic $\left(\text{Δ}E_{IJ}^{ES}\right)$ and charge transfer with higher-order mixed terms energies $\left(\text{Δ}E_{IJ}^{CT+mix}\right)$, while hydrophobic interaction can be suggested by dispersion $\left(\text{Δ}E_{IJ}^{DI}\right)$. The steric repulsion between atoms was described by the exchange-repulsion energy $\left(\text{Δ}E_{IJ}^{EX}\right)$, while the water mediated interaction can be described by solvation energy $\left(\text{Δ}E^{Gsol}\right)$. PIEDA showed that the phenyl ring or the bicyclic core of the fragments interact with residues αPhe255 and αPhe257 through hydrophobic interaction (Fig. 3A, B, C, D and E). These residues belong to the FPF loop, which is conserved across all bacterial species [58] (Supplementary Fig. S10). Each of the three aromatic interactions makes edge-to-face contacts. The distance between the centroid of the ligand phenyl ring and centroid of the phenyl rings of αPhe255 and αPhe257 and that between the αPhe255/αPhe257 ligand is ~5–5.5 Å. These values are in agreement with the values obtained from various protein structures whose phenyl ring centroids are commonly separated by about 5.5 Å [59]. Also, the dihedral angles within this network are close to 90^◦^, consistent with the values observed in previous studies. Residues αThr258 and αAla305 make hydrophobic interactions with the phenyl core of the fragments D-116, D-004 and D-735. In addition, a strong hydrogen bond between NH2 of D-116 and αGln215 is observed and, a proton of the protonated N of the benzoimidazole core interacts with αGlu217 through salt bridge interaction (Fig. 3A). FMO analysis showed a strong hydrogen bond between the proton of NH2 moiety in D-004 and residue αGlu217 (Fig. 3B). One of two carbonyl groups of the D-555 interacts with αGln215 and αGly282 through hydrogen bonds. The core and the carbonyl oxygen of the oxazinone moiety interacts with αSer177 and αArg201 through hydrogen bonds, respectively (Fig. 3C). The FMO analysis indicates that the sulphonamide group of the D-735 interacts with αArg201 and αGln158 through strong hydrogen bonds. Also, the aniline nitrogen form hydrogen bond with the αGlu217 of D-735 fragment (Fig. 3D). A strong Π-cation interaction between the imidazolidinedione core of D-876 and αArg201 is observed. The carbonyl oxygen of the imidazolidinedione core interacts with αGln215 of *Mt*PheRS through hydrogen bond (Fig. 3E).

To investigate whether the fragments occupying the L-Phe binding site of *Mt*PheRS can inhibit its function, we tested L-Phe adenylation in presence of fragments at 3- and 4.5-mM concentrations respectively. The reaction mixture contained unmodified *Mt*tRNA^Phe^ transcripts and 5 and 10 % DMSO. Although *Mt*PheRS can activate phenylalanine in the absence of tRNA^Phe^, our earlier studies demonstrated that addition of tRNA^Phe^ stimulate this reaction [17]. The activity of *Mt*PheRS in the presence of 5 % DMSO was comparable with activity observed in the absence of DMSO (Fig. 3F). The presence of 10 % DMSO decreased Phe-AMP synthesis. Interestingly, 10 % DMSO increased L-Phe adenylation in the presence of the very hydrophobic D-116 fragment. Synthesis of L-Phe-AMP was inhibited the least by D-735 fragment (Supplementary Fig. S11).

### 3.5. Comparison of HsPheRS and MtPheRS

The L-Phe bound heterotetrameric structure of cytoplasmic *Homo sapiens* (*Hs*) (PDB ID: 3L4G) and *Mt*PheRS (PDB ID: 7KA0) were found to be similar, with all containing two α and two β subunits. The α subunits of *Hs* and *Mt*PheRS share 30 % overall sequence similarity (Supplementary Fig. S10) and show rmsd of 1.8 Å for 200 ${\text{C}}_{\text{α}}$ atoms after superimposition. The catalytic core α2 domain of both enzymes share similar fold and secondary structural elements, while the N-terminal α1 domain diverges significantly (Supplementary Fig. S12). Close inspection of the active site residues in the α2 domain, indicates that all five fragments could be potentially accommodated in the L-Phe binding site of *Hs*PheRS. The side chains of residues in the L-Phe binding pocket of *Hs*PheRS are found in close proximity to the fragments (Supplementary Fig. S13). The bacterial conserved FPF loop comprising residues αPhe255 and αPhe257 is substituted by residues αAsn410 and αTyr412 respectively in eukaryotes. The phenyl ring of the L-Phe substrate is anchored by the edge-to-face interactions with aromatic rings of αTyrα412 and αPhe438 residues in the *Hs*PheRS structure. Furthermore, the residues in the *Mt*PheRS that interact with the fragments are conserved in the *Hs*PheRS (Supplementary Fig. S10). This observation indicates that the fragments need to be further modified to improve their selectivity over the human counterpart. This is the expected result, as the fragment library screening only identifies small chemical moieties that need further design to yield potent and selective inhibitors.

### 3.6. Post-transfer editing complex of PheRS/tRNA^Phe^

**Fig. 4.**
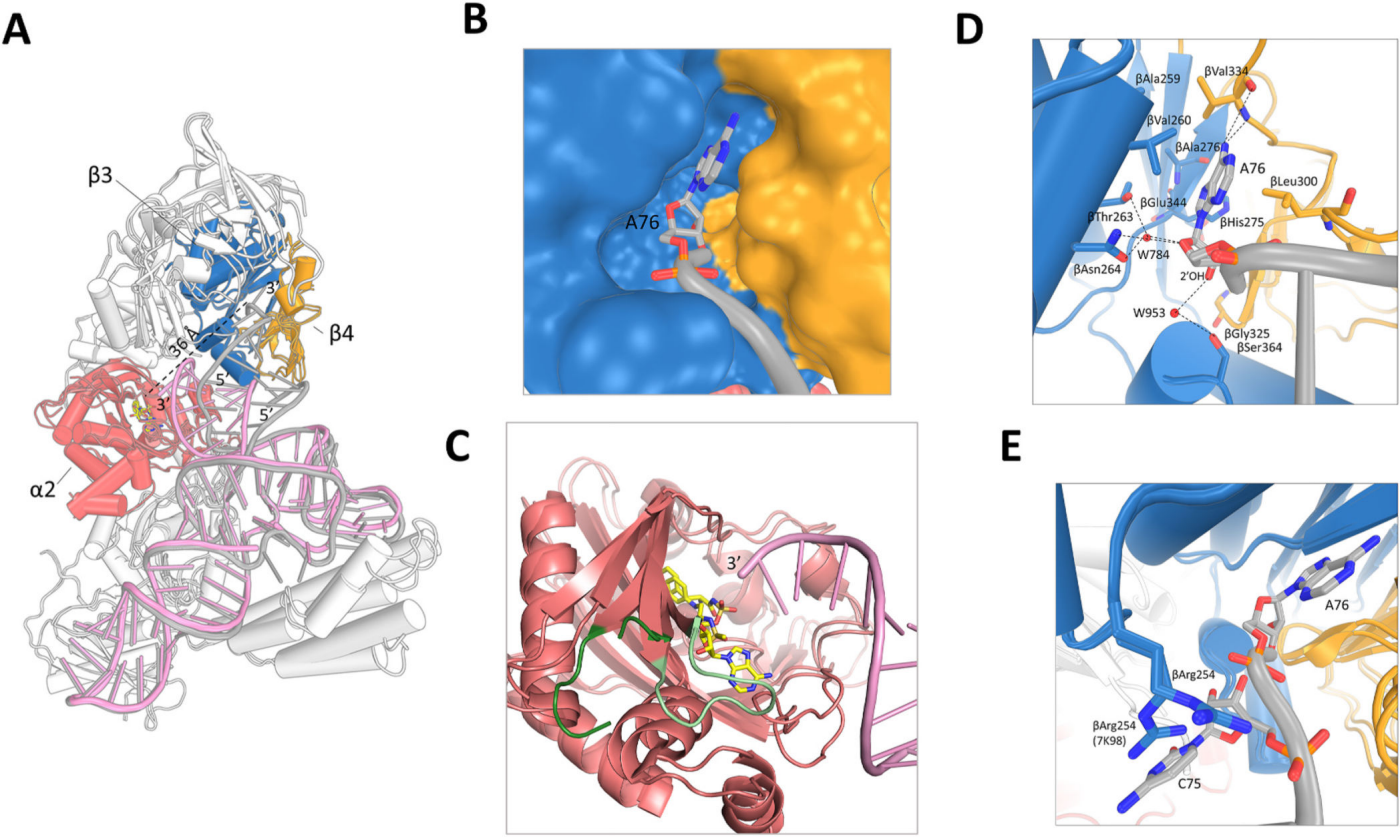
(**A**) Superimposition of Phe-AMS bound aminoacylation ready state (PDB ID:7K98) and D-735 bound heterodimeric *Mt*PheRS/tRNA^Phe^ structures. Both the Phe-AMS and D-735 in the synthetic site are shown as yellow sticks. (**B**) Surface view representation of β3/4 interface at the editing site. (**C**) Superimposition of the α2 domain of the synthetic site Phe-AMS bound aminoacylation ready state and the D-735 bound structures of *Mt*PheRS/tRNA^Phe^. The β-hairpin in the synthetic site of the Phe-AMS bound aminoacylation ready state and the D-735 bound *Mt*PheRS/tRNA^Phe^ structures is colored as light and dark green, respectively. The Phe-AMS and D-735 is depicted as stick model. (**D** and **E**) Detailed view of editing site residues interacting with the 3′ CCA end of the tRNA^Phe^ in the D-735 bound *Mt*PheRS/tRNA^Phe^ complex structure. The *Mt*PheRS of Phe-AMS bound aminoacylation ready state structure is shown in gray and its tRNA^Phe^ is colored as pink. The D-735 bound *Mt*PheRS/tRNA^Phe^ structure is shown in gray except α2, β3, β4 domains and tRNA^Phe^ are colored same as shown in Fig. 2. The protein residues and the terminal A76 nucleotide of tRNA^Phe^ are shown as sticks. Water molecules are illustrated as red spheres. Hydrogen bond is shown as dashed lines. (For interpretation of the references to color in this figure legend, the reader is referred to the Web version of this article.)

In one of the αβ heterodimer of the *Mt*PheRS/tRNA^Phe^ ${\left(\text{αβ}\right)}_{2}$ complex bound to D-735, clear electron density directed towards the β3 and β4 domains of the β subunit was observed. We modeled the 3′ CCA end of the tRNA^Phe^ acceptor stem into this density (Fig. 4A). An electron density that may be unambiguously attributed to the terminal A76 of the tRNA^Phe^ was present in the deep, narrow tunnel that forms at the interface between the β3 and β4 domains of the β subunit (Fig. 4B). The β3/4 domain is composed of a central four-stranded antiparallel β sheet and two α helices sandwiched between the seven stranded β sheet and two α helices. The editing site of bacterial PheRS is known to be localized at the interface between β3 and β4 domains [16]. This structure corresponds to the post-transfer editing state of the enzyme, where the mischarged Tyr-tRNA^Phe^ is displaced from aminoacylation site for proofreading. The D-735 compound occupying the synthetic site is separated by ~36 Å from acceptor end of tRNA^Phe^ within the editing site (Fig. 4A). This observation is in agreement with the predicted post-transfer editing model of class II aaRSs, suggesting that bending of the 3′ tRNA end alone would be sufficient to switch the 3′ CCA charged with amino acids between the active and editing sites [16,60]. Comparison of the D-735 bound *Mt*PheRS/tRNA^Phe^ structure with the Phe-AMS bound aminoacylation ready state structure (PDB ID: 7K98) shows an outward movement of the flexible disordered β-hairpin (residues 268–276) in the α2 domain (Fig. 4C). The β-hairpin is observed to be ordered in the Phe-AMS bound aminoacylation ready state structure of *Mt*PheRS/tRNA^Phe^. This observation suggests that, in the presence of L-Phe in synthetic site, the β-hairpin opens to accommodate ATP. Upon activation of L-Phe, the β-hairpin moves closer to the synthetic site and stabilize the AMP moiety. This conformation of *Mt*PheRS can accommodate the 3′ CCA end of tRNA^Phe^ in the α2 domain, as observed in the Phe-AMS bound aminoacylation ready state structure. In all the fragment bound and the L-Phe bound *Mt*PheRS/tRNA^Phe^ (PDB ID: 7KA0) structures, the β-hairpin is positioned outward, suggesting an open conformation of the α2 domain and the 3’ CCA end of the tRNA^Phe^, most likely to be inserted in the editing domain (Supplementary Fig. S14). However, we were able to capture such conformation in only one of the αβ heterodimer *Mt*PheRS/tRNA^Phe^ bound to D-735 fragment.

A remarkable structural peculiarity is the rotational positioning of the terminal A76 adenosine moiety of tRNA^Phe^. The A76 adenine ring is stabilized in the hydrophobic pocket comprising residues βAla259, βVal260, βAla276, βLeu300 and βVal334 (Fig. 4D). The plane of the A76 base occupies a position midway between these residues. Residues βAla259 and βVal260 belong to the conserved motif of domain β3, while residue βLeu300 is localized in the β-hairpin (residues 294–307) of the β4 domain (Supplementary Fig. S15). A critical role of βLeu300 toward the editing activity of PheRS has been confirmed by kinetic and mutagenesis studies in PheRS from *Pyrococcus horikoshii* [33]. The imidazole ring of the invariant βHis275 shows stacking interaction with the adenine moiety. The orientation of the ribose is such that the mischarged Tyr attached to the 2′-OH can be accessible in the editing site for hydrolysis. The tyrosyl moiety, if attached to the 3′-OH of the ribose could possibly cause steric clash with C75 of the tRNA^Phe^. The bacterial PheRS is an exception in the class IIa aaRSs due to its aminoacylation activity on the 2′-OH of the ribose [61]. Our previous structural studies confirmed this activity for L-Phe [16] and suggests that similarly to the cognate substrate, bacterial PheRS should catalyze the attachment of Tyr moiety at the 2′-OH of the ribose of A76. Furthermore, the conserved βGlu344 and βGly325 (βGlu334 and βGly315 from *Thermus thermophilus* PheRS (*Tt*PheRS)) residues, involved in the specific recognition and anchoring of the Tyr moiety in the editing site [16] should be in close proximity to tyrosine only when it is attached at the 2′-OH position of the ribose. Previous studies have suggested the role of conserved βThr263, βAsn264 and βSer364 (corresponding to βThr249, βAsn250 and βThr354 in *Tt*PheRS) residues in positioning the catalytic water molecules for cleaving the ester bound between 2′ hydroxyl group of adenosine and the carboxyl group of L-Tyr [62,63]. In our structure, these residues are close with the ribose moiety (Fig. 4D). The side chain oxygen atoms of both the βThr263 and βAsn264 interact with the water S784 through hydrogen bond interaction. Also, another weak hydrogen bond interaction is observed between the side chain nitrogen atom of βAsn264 and the O4′ atom of ribose. Based on this observation, we postulate that in the presence of mischarged L-Tyr, the ribose moiety of A76 could potentially reorient in such a configuration that the ester bond is positioned close to the catalytic water molecule. In addition, the 2′-OH group of the ribose interacts with the βSer364 through water (W953) mediated hydrogen bonds (Fig. 4D). The W953 molecule can possibly interact with the aminoacyl ester bond during hydrolysis. A recent crystal structure of *Pyrococcus abyssi* ThrRS has also unveiled an interaction between the 2′-OH and a potential catalytic water molecule [64]. The accurate positioning of the 3′ CCA end of acceptor arm into the editing site is assisted by a salt bridge interaction between βArg254 and C75 nucleotide (Fig. 4E). When compared with the structure of *Mt*PheRS in the aminoacylation ready state (PDB ID: 7K98), a rotamer switch of βArg254 is observed to avoid the steric clash with the tRNA^Phe^ in the editing site (Fig. 4E). This suggests that the βArg254 can act as a lid to prevent the binding of Phe-tRNA^Phe^ or be open to allow Tyr-tRNA^Phe^ binding to editing site. This observation is in agreement with the previous studies, where βArg244 of *Tt*PheRS interacts with the C75 nucleotide of the tRNA^Phe^ under oxidation stress [63].

### 3.7. Structural comparison of editing site of PheRS

**Fig. 5.**
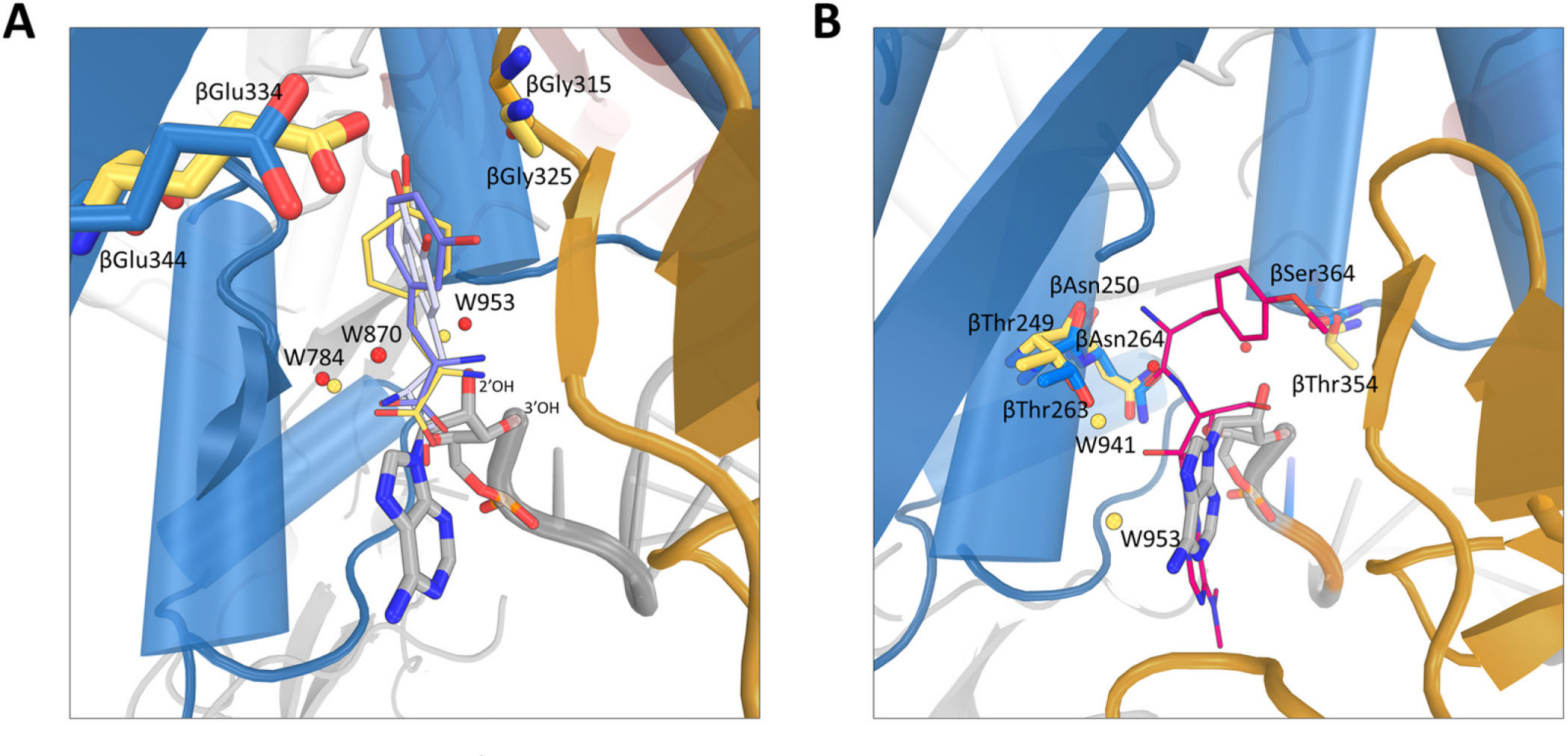
Superimposition of D-735 bound *Mt*PheRS/tRNA^Phe^ complex structure with (**A**) L-Tyr (yellow colored; PDB ID: 2AMC), meta-Tyr (violet colored; PDB ID: 3HFZ), L-Dopa (light gray colored; PDB ID: 3TEH) and (**B**) puromycin (magenta colored; PDB ID: 4TVA) bound *Tt*PheRS structures. Water molecules in the *Mt* and *Tt* structures are shown as red and yellow spheres respectively. Residues of *Tt* and *Mt*PheRS are shown as yellow and blue sticks respectively. (For interpretation of the references to color in this figure legend, the reader is referred to the Web version of this article.)

Structural comparison of the editing sites of *Tt*PheRS complexed with L-tyrosine (PDB ID: 2AMC), meta-tyrosine (PDB ID: 3HFZ), and L-Dopa (PDB ID: 3TEH), with *Mt*PheRS/tRNA^Phe^/D-735 complex reveals the proximity of the tyrosine ligands with the terminal A76 of the tRNA^Phe^ [16,65,66] (Fig. 5A). The aromatic rings of L-tyrosine, meta-tyrosine, and L-Dopa show slight rotation and positional shift relative to each other. In the L-tyrosine bound *Tt*PheRS structure, the conserved residues βGlu334 and βGly315, which plays a critical role in specific recognition of the Tyr are closely approximating in space of the residues βGlu344 and βGly325 of the *Mt*PheRS. Also, the 2′-OH of the ribose is in proximity with the main chain carboxyl and the amino group of the tyrosine amino acid. The appearance of A76 at the editing site is accompanied by loss of water molecule when compared with the tyrosine bound structure of PheRS. The water molecule at position 870 is close to the tyrosine moiety and therefore, might be displaced or lost in presence of mischarged Tyr in the editing site (Fig. 5A). Interestingly, both the positions of potential catalytic water molecules (W784 and W953) in the *Mt*PheRS/tRNA^Phe^/D-735 complex are preserved (W1087 and W1075 in *Tt*PheRS) in the tyrosine bound structure of *Tt*PheRS (Fig. 5A). Altogether, it is therefore reasonable to assume that the editing state of *Mt*PheRS in comparison with the Tyr bound *Tt*PheRS demonstrates the closest picture of the transition post-transfer state, where the mischarged Tyr is removed from 3′end of the tRNA^Phe^.

Comparison with the puromycin bound structure of *Tt*PheRS (PDB ID: 4TVA) shows that the adenine moiety of A76 of tRNA^Phe^ is in plane with the modified adenine of the puromycin (Fig. 5B). However, there is a slight position and rotation shift of the ribose relative to each other. These differences could be related to the 3′-O-aminoacylated isomeric form of puromycin in *Tt*PheRS. The conserved βThr263, βAsn264 and βSer364 of *Mt*PheRS align well with the βThr249, βAsn250 and βThr354 residues in the *Tt*PheRS. However, the proposed catalytic water molecules W1087 and W1075 in *TtPheRS* (W784 and W953 in *Mt*PheRS) were found to be missing in the puromycin bound *Tt*PheRS structure (Fig. 5B). The presence of puromycin in the editing site was accompanied by presence of water molecules (W941 and W953) at different position, which were proposed to catalyze the hydrolysis of ester bond (Fig. 5B) [62]. However, both these water molecules are found to be missing in the *Mt*PheRS structure.

## 4. Discussion

Heterocyclic quinoline and quinazoline compounds show great interest in the area of medicinal chemistry due to their wide range of antibacterial, antifungal [67], antiviral [68], anti-inflammatory [69] activities. These compounds have been explored as a promising scaffold against tuberculosis [70]. The benzimidazole moiety is used as scaffolds to synthesize selective drugs of interest in various therapeutic areas [71, 72]. Furthermore, various derivatives of 1,4-benzoxazin have been shown to possess promising antimicrobial activity [73]. The 1,4-benzoxazin scaffold show their inhibitory effects against the poly (ADP-ribose) polymerase enzyme [74]. Using NMR and SPR studies, we have identified derivatives of quinoline, quinazoline and benzimidazole which bind *Mt*PheRS. Further, by performing crystallographic studies, we have investigated the molecular basis of *Mt*PheRS binding to these fragments. The bicyclic quinoline, quinazoline and benzimidazole moieties of the fragments are completely enclosed in the L-Phe binding site.

**Fig. 6.**
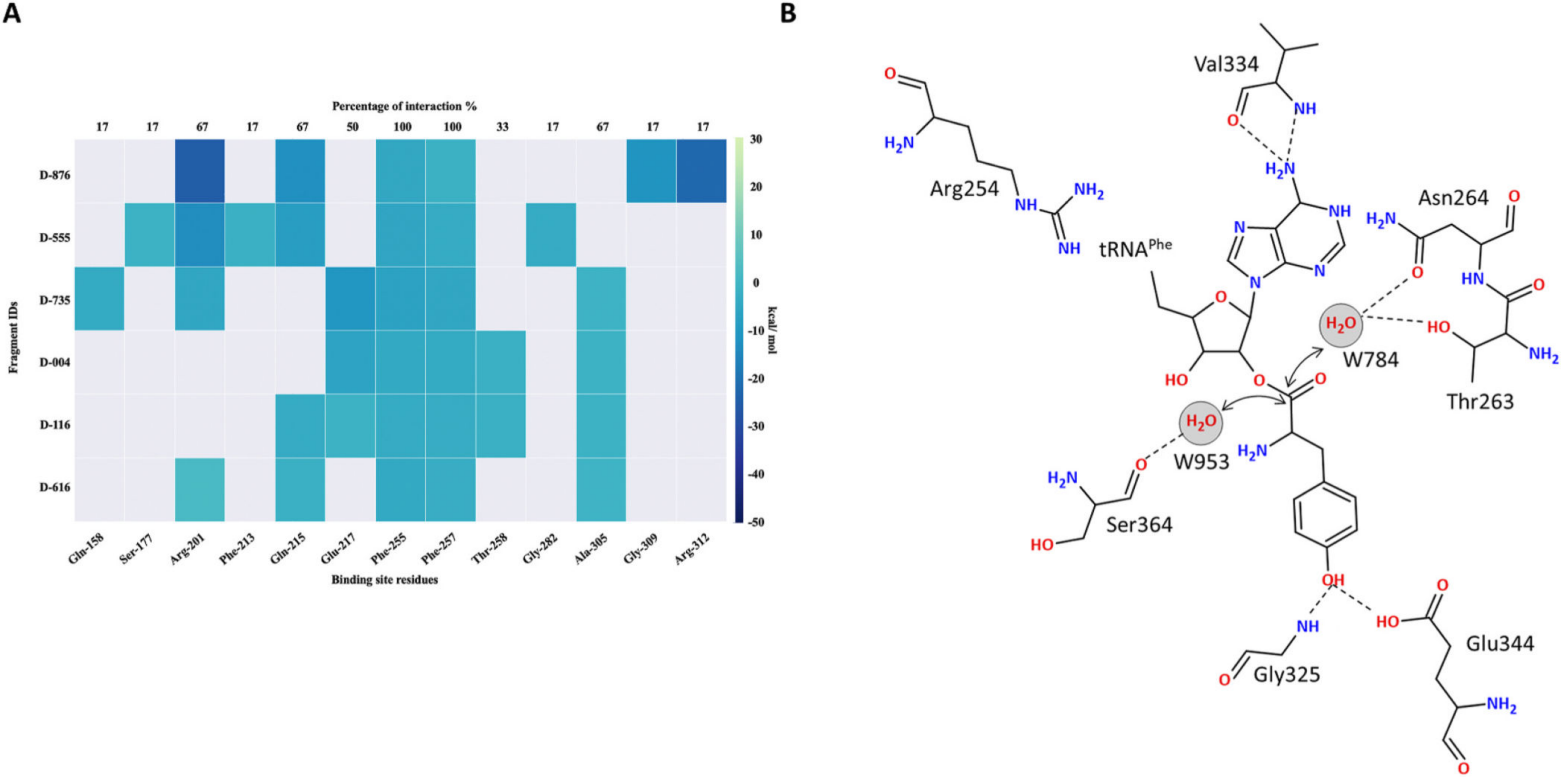
(**A**) Fragment-protein interaction energy heat map (**B**) Proposed mechanistic model for Tyr-tRNA^Phe^ hydrolysis at the editing site of *Mt*PheRS.

The phenylalanine binding pocket of *Mt*PheRS consists of residues that can contribute to hydrophobic interactions (αPhe255, αPhe257, αThr258 and αAla305) and capable of engaging in hydrogen bonding and electrostatic interactions (αGln215, αGlu217, αGly309, and αArg312 on the right side, and αGln158, αArg201, and αSer177 on the left side). Although these five fragments represent different chemical scaffolds, all show a consistent binding mode, which is stabilized through a combination of polar and hydrophobic interactions with above listed residues. The L-Phe site shows significant flexibility in accepting several chemical scaffolds. In contrast none of these molecules were found in the editing site on β subunit. To identify and investigate the hotspot residues in the fragment binding site of α subunit, we determined the binding interaction energies based on the FMO analysis. The *Mt*PheRS/fragment interaction heat map shows overall conserved patterns for the hydrophobic interactions with αPhe255 and αPhe257 (Fig. 6A). Hydrophobic interactions with αAla305 are retained in fragments D-735, D-004 and D-116. The top-ranking fragment D-876 maintains all the critical interactions and makes additional interactions with residues αArg201, αGln215, αGly309 and αArg312. In accordance with this, binding of D-876 fragment exhibited highest interaction energy (−93.6 kcal/mol; Table 2) and ligand efficiency score (5.2; Table 2). Fragment D-555 is unique in forming key interactions with αSer117, αPhe213 and αGly282 residues. This information about interaction energies at per-residue level will help in fragment growing during the compound optimization process. Overall, these fragments serve as an excellent starting point for the development of novel antitubercular treatments through a structure-guided drug discovery approach. Thus, we envisage that the potency of these fragments can be improved by extending towards the more hydrophilic ATP binding pocket or an auxiliary hydrophobic pocket next to the phenylalanine binding site.

### 4.1. Structural comparison with other inhibitor-bound structures of PheRS

Previous crystallographic studies of bacterial PheRS have revealed the presence of an auxiliary hydrophobic pocket adjacent to the L-Phe and ATP binding site of α subunit [29,30]. This auxiliary pocket is comprised of mainly hydrophobic residues, including αPhe143, αPhe148, αAla154, αHis175, αPhe254 and αPro256 (Supplementary Fig. S16). Phenyl-thiazolylurea-sulfonamides inhibitors of PheRS extend into the auxiliary pocket of PheRS along with the L-Phe binding site. Also, a bicyclic derivative of azetidines inhibits *Plasmodium falciparum* cytosolic PheRS (*Pf*PheRS) by binding at both the L-Phe and auxiliary pocket of *Pf*PheRS [75]. Among the compounds specifically inhibiting *Mt*PheRS, the GDI05–001 binds to L-Phe, ATP as well as auxiliary pocket of the protein [30] (Supplementary Fig. S16). The indole moiety of GDI05–001 is bended almost vertically to the phenylsulfonamide and projects into the hydrophobic auxiliary pocket. However, the auxiliary pocket in the structures of PheRS with bound fragments was completely unoccupied offering an opportunity for fragment growth by adding hydrophobic substituents to explore the auxiliary pocket of *Mt*PheRS.

### 4.2. Mechanistic model of post-transfer editing by PheRS

Previous structural modeling and mutational studies of the editing activity of PheRS postulated the involvement of water molecules as catalytic agents in ester bond hydrolysis [16,63]. Our structural data of one of the αβ heterodimer of *Mt*PheRS/tRNA^Phe^ bound to D-735 provides the experimental validation of the water-mediated hydrolytic mechanism of ester cleavage in the editing site. Based on the arrangement of the CCA arm of tRNA^Phe^ in the editing site, we have proposed a mechanistic model for post-transfer editing activity by PheRS (Fig. 6B). Upon the activation of tyrosine to yield Tyr-tRNA^Phe^ in the synthetic site, the CCA arm of the mischarged tRNA^Phe^ translocate at the interface between β3/4 domain of the β subunit of the heterotetramer. This repositioning is orchestrated by the interaction of βArg254 with the C75 nucleotide of tRNA^Phe^. Further, Tyr attached A76 substrate rotates towards the hydrophobic pocket. This configuration juxtaposes the ester bond in proximity to two catalytic water molecules (W784 and W953). The editing specificity is ensured by hydrogen bonding interaction between the hydroxyl group of Tyr and the side chain carbonyl and amino groups of βGlu344 and βGly325 respectively. The hydrolytic reaction proceeds with a nucleophilic attack on the ester bond catalyzed by water molecule W784, precisely aligned by βThr263 and βAsn264 residues. Simultaneously, water molecule W953, hydrogen bonded with βSer364, acts as a proton donor, stabilizing the leaving group and thus completing the reaction. This mechanism highlights the enzyme’s primary function to provide specificity rather than catalyze the hydrolysis. Our proposed model aligns with prior functional studies, where the editing activity of PheRS was impacted moderately upon substituting residues within the editing site [63].

Previous studies examining the editing sites of LeuRS, IleRS, and ValR have highlighted the significance of water molecules interacting with the 3′-OH group of terminal adenosine [76,77]. Also, studies on the editing process of PheRS highlighted the critical role of the 3′-OH moiety of A76 towards the activating of the catalytic water molecule [63]. However, in contrast to these studies, our structure analysis reveals no interaction of the 3′-OH moiety with any water molecules. In summary, this study allows a comprehensive understanding of the post-transfer editing activity of PheRS, which provides new insight into the mechanism of proof reading in class II synthetases.

## Supplementary Material

MtbPheRS_FragScreening

PheRS_FraScr_Fig.S16

Appendix A. Supplementary data

Supplementary data to this article can be found online at https://doi.org/10.1016/j.ejmech.2025.117335.

## Figures and Tables

**Table 1:** Data collection and refinement statistics ^a^.

Fragment	D-555	D-876	D-735	D-116	D-004
PDB ID	9DSX	9DTF	9DRT	9DRS	9DRV
**Data collection statistics**					
Wavelength (Å)	0.9792	0.9794	0.9794	0.9792	0.9792
Resolution (Å)	2.05–50.00 (2.05–2.09)	2.45–50.00 (2.45–2.49)	2.51–50.00 (2.51–2.55)	2.35–50.00 (2.39–2.35)	2.45–50.00 (2.49–2.45)
Cell parameters (Å, °)	*a* = 147.3, *b* = 65.03, *c* = 189.5, β = 110.9	*a* = 146.7, *b* = 64.07, *c* = 187.9, β = 110.9	*a* = 147.8, *b* = 65.25, *c* = 191.8, β = 109.7	*a* = 146.7 *b* = 64.11 *c* = 188.3, β = 111.2	*a* = 147.1, *b* = 64.39, *c* = 188.8, β = 111.1
Space group	P2_1_	P2_1_	P2_1_	P2_1_	P2_1_
Unique reflections (merged)	208,786 (10,395)	124,143 (6140)	117,034 (5751)	135,377 (6682)	119,855 (5966)
Completeness (%)	99.3 (99.0)	99.5 (99.5)	98.4 (98.3)	99.5 (99.5)	99.2 (99.3)
Multiplicity	6.6 (6.0)	15 (9.2)	6.6 (6.6)	6.4 (4.9)	4.2 (3.9)
Mean I/sigma	28.3 (2.7)	11.6 (1.4)	18.6 (1.8)	16.5 (1.6)	10.9 (1.3)
R_merge_^b^	0.063 (0.911)	0.149 (1.354)	0.092 (1.040)	0.111 (0.972)	0.126 (1.211)
CC1/2 ^c^	0.998 (0.704)	0.997 (0.579)	0.996 (0.744)	0.99 (0.56)	0.97 (0.52)
**Refinement statistics**					
Resolution range (Å)	2.05–48.86	2.45–48.64	2.51–48.42	2.35–46.77	2.46–48.75
Reflections working/test	198,550/9901	108,823/5527	110,669/5476	127,062/7791	108,242/5431
*R*_work_/*R*_free_ (%)^d^	20.0/23.3	20.3/24.9	20.7/24.1	20.2/24.9	20.7/25.9
Number of atoms	22,325	21,922	22,065	21,277	21,397
protein atoms	17,634	17,610	17,733	17,339	17,452
RNA atoms	3126	3126	3169	3045	3045
ligand atoms	209	222	164	170	152
water atoms	1356	964	999	723	748
Root mean square deviation					
bond lengths (Å)	0.002	0.002	0.002	0.002	0.002
angles (°)	0.501	0.403	0.426	0.46	0.44
Ramachandran plot					
favored	97.1	96.3	96.9	96.5	96.1
outliers	0.31	0.31	0.22	0.39	0.3
MolProbity clashscore	2.60	3.87	3.43	4.38	5.1
Average B factor					
all atoms (Å^2^)	40.22	51.84	55.56	53.15	55.53
protein atoms (Å^2^)	35.05	47.05	49.38	49.57	51.49
RNA atoms (Å^2^)	73.02	83.67	95.47	78.21	82.66
ligand atoms (Å^2^)	42.78	51.65	52.06	45.85	51.98
solvent atoms (Å^2^)	31.43	36.09	39.27	35.12	40.05

a Values in parentheses correspond to the highest resolution shell.

b ${\text{R}}_{\text{merge}}=\text{ΣhΣj}|\text{Ihj}-<\text{Ih}>|/\text{ΣhΣjIhj}$, where Ihj is the intensity of observation j of reflection h.

c CC1/2 As defined by Karplus and Diederich.

d R=Σh|Fo|−|Fc|/Σh|Fo| for all reflections, where Fo and Fc are observed and calculated structure factors, respectively. Rfree is calculated analogously for the test reflections, randomly selected, and excluded from the refinement.

**Table 2:** Ligand interaction energy and ligand efficiency scores.

Fragment	Interaction Energy (kcal/mol)	LEI	Rank Order
D-876	−93.6	93.6/18 = 5.2	1
D-735	−61.7	61.7/14 = 4.4	2
D-555	−64.0	64/18 = 3.5	3
D-004	−41.4	41.4/11 = 3.7	4
D-116	−37.0	37.0/10 = 3.7	5

## Data Availability

No data was used for the research described in the article.
