# Supplementary material for: Different chemical scaffolds bind to L-phe site in Mycobacterium tuberculosis Phe-tRNA synthetase
Source: Eur J Med Chem. Author manuscript; Available in PMC 2025 Sep 19. (PMC12447630; doi:10.1016/j.ejmech.2025.117335)
Supplement: MtbPheRS_FragScreening [file NIHMS2096802-supplement-MtbPheRS_FragScreening.pdf]

SUPPORTING INFORMATION

Different chemical scaffolds bind to L-Phe site in *Mycobacterium tuberculosis* Phe-tRNA synthetase

Priyanka Gade[1,2], Changsoo Chang[1,2], Denise S Pryde[3], Daniel Fletcher[3], Sarah Niven[3], Luma Godoy Magalhaes[3], David Robinson[3], Jagmohan Saini[3], Peter E. G. F. Ibrahim[3], Barbara Forte[3], Jacek Wower[4], Michael J. Bodkin[3], Beatriz Baragaña[3*], Ian H. Gilbert[3], Karolina Michalska[1,2], and Andrzej Joachimiak[1,2,5*]

[1] Center for Structural Biology of Infectious Diseases, Consortium for Advanced Science and Engineering, University of Chicago, Chicago, Illinois 60667, USA

[2] Structural Biology Center, X-ray Science Division, Argonne National Laboratory, Lemont, Illinois 60439, USA

[3] Drug Discovery Unit, Wellcome Centre for Anti-Infectives Research, Division of Biological Chemistry and Drug Discovery, University of Dundee, Dundee, DD1 5EH, UK

[4] Department of Animal Sciences, Auburn University, Auburn, AL 36849, USA

[5] Department of Biochemistry and Molecular Biology, University of Chicago, Chicago, IL, 60367, USA

* To whom correspondence should be addressed. Tel: +1 630 252 3926; Fax: +1 630 252 6126; Emails: andrzejj@anl.gov and B.Baragana@dundee.ac.uk

Present address: Andrzej Joachimiak, Structural Biology Center, X-ray Science Division, Argonne National Laboratory, Lemont, Illinois 60439, USA and Beatriz Baragaña, Drug Discovery Unit, Wellcome Centre for Anti-Infectives Research, Division of Biological Chemistry and Drug Discovery, University of Dundee, Dundee, DD1 5EH, UK

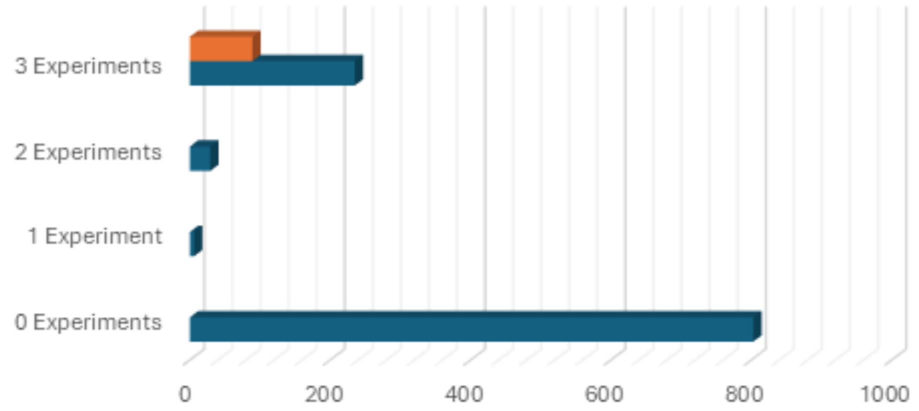

	0 Experiments	1 Experiment	2 Experiments	3 Experiments
Fragments showing competiton				89
# of Fragments positive in experiment	803	6	29	235

Fragments showing competiton   # of Fragments positive in experiment

Figure S1. NMR fragment screening summary. The number of fragments from the screen which showed evidence of binding in 0, 1, 2 or 3 of the experiments in blue with the number of fragments which show competition with addition of known binder in orange.

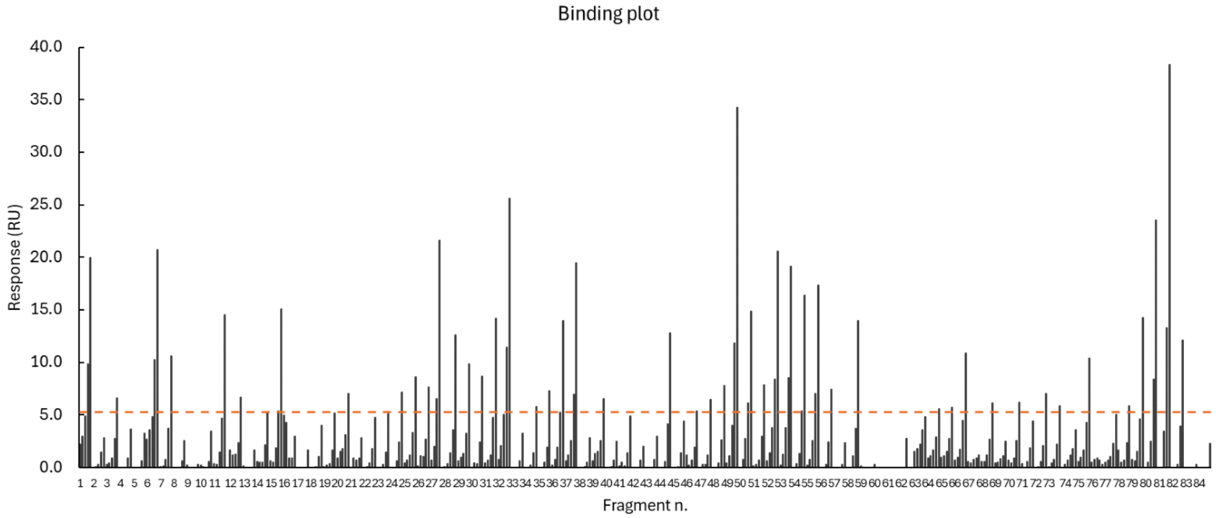

Figure S2. Representative plot for qualitative analysis of binding between fragments and *Mt*PheRS from SPR. Binding cut-off was established using the binding level screen boundary tool implemented on the Biacore™ Insight Evaluation Software 5.0.18.22102. First concentration of each fragment series is represented by numbers on *x*-axis and level of response (resonance units) can be seen on *y*-axis. Binding level was adjusted by blank injection subtraction. Cut-off for binding is represented by the horizontal dotted line.

DDD00005864

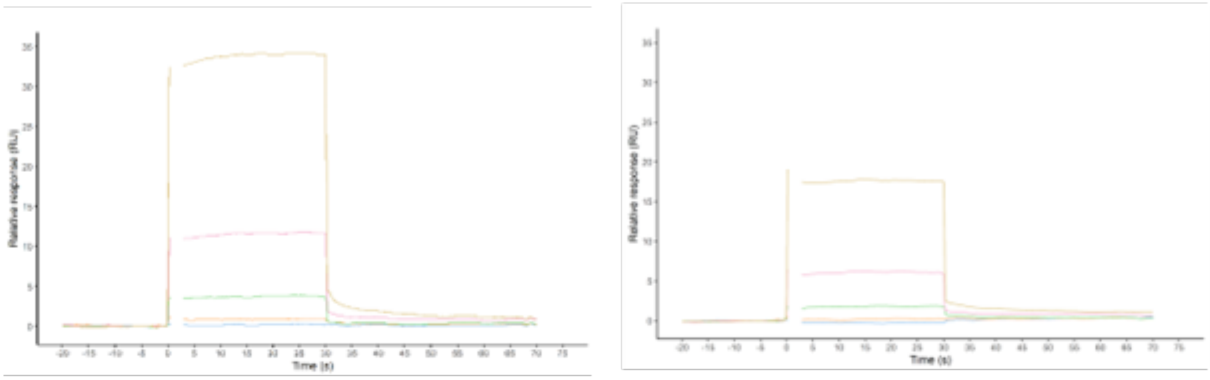

DDD00072751

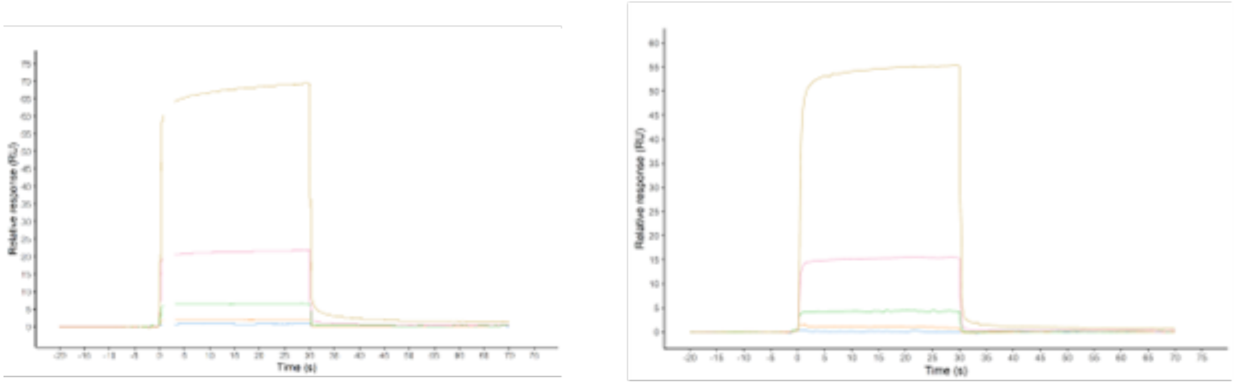

DDD00079004

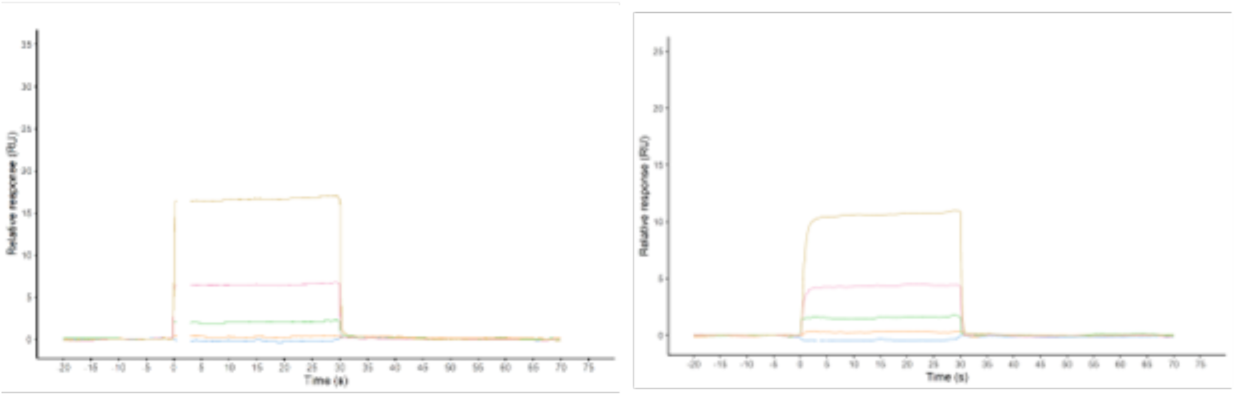

DDD00100406

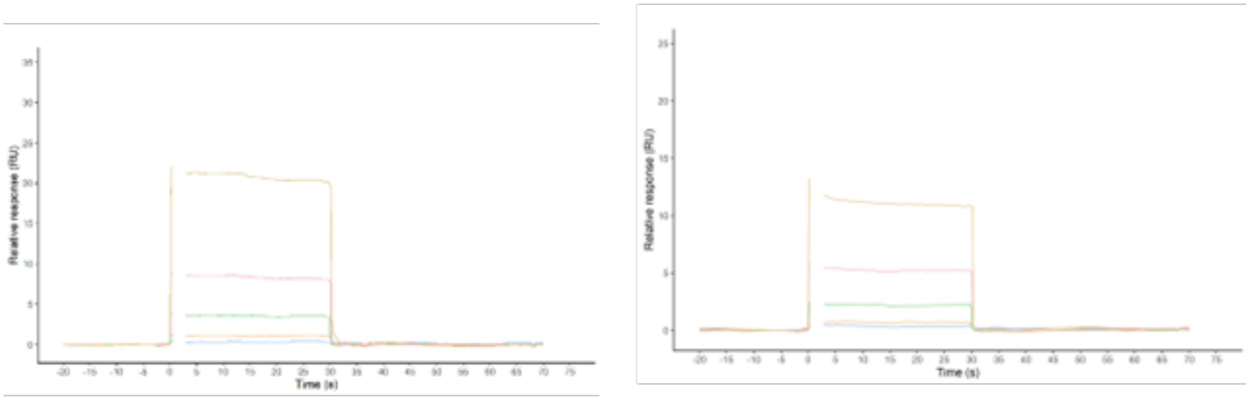

DDD00100593

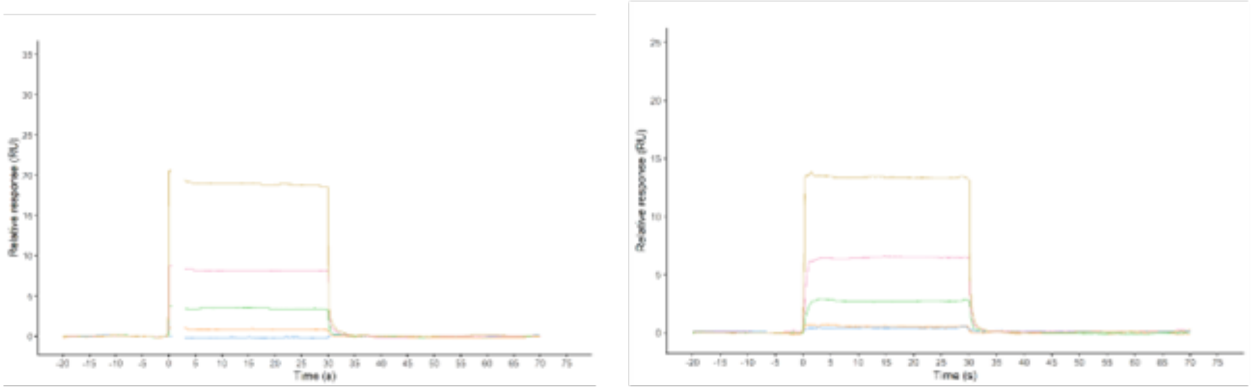

DDD01008820

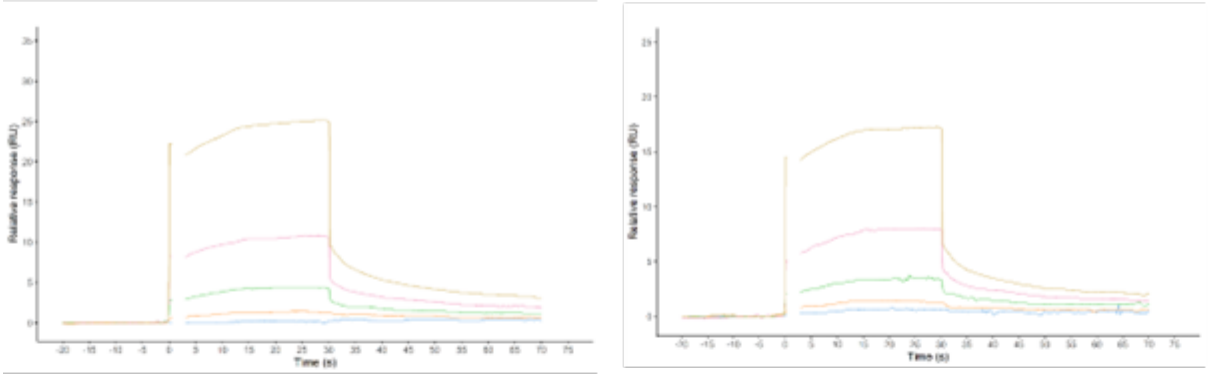

DDD01305584

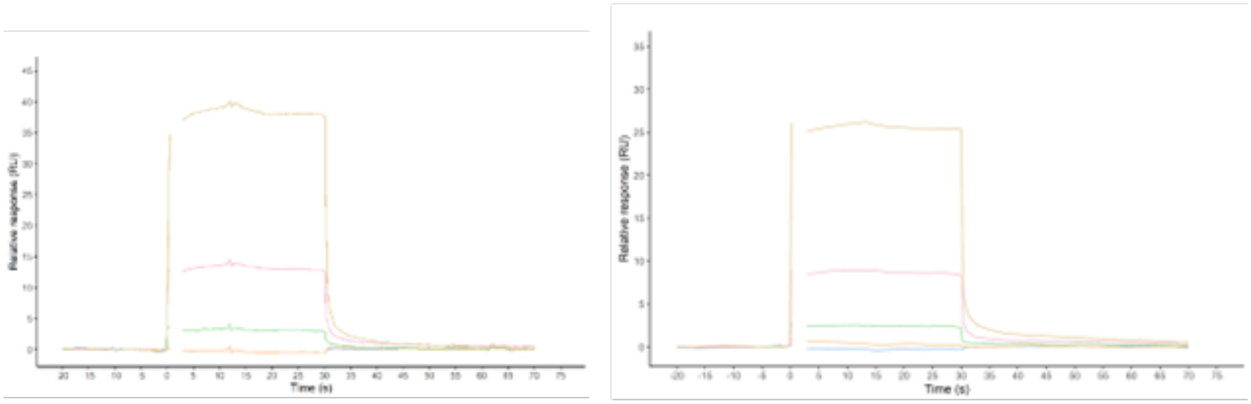

DDD01305616

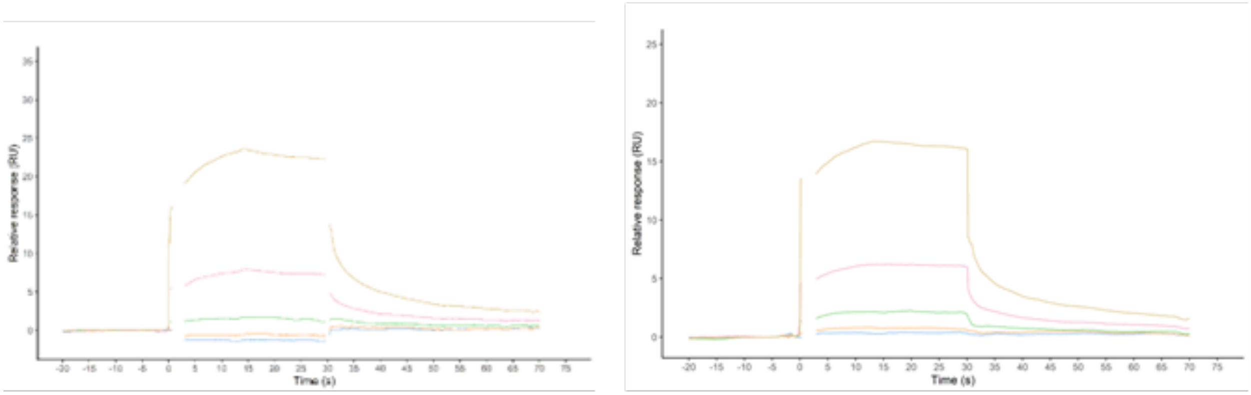

Figure S3. Kinetic plots for each fragment binding to *Mt*PheRS. Time (in seconds) can be seen on x-axis and level of response (resonance units) can be seen on y-axis.

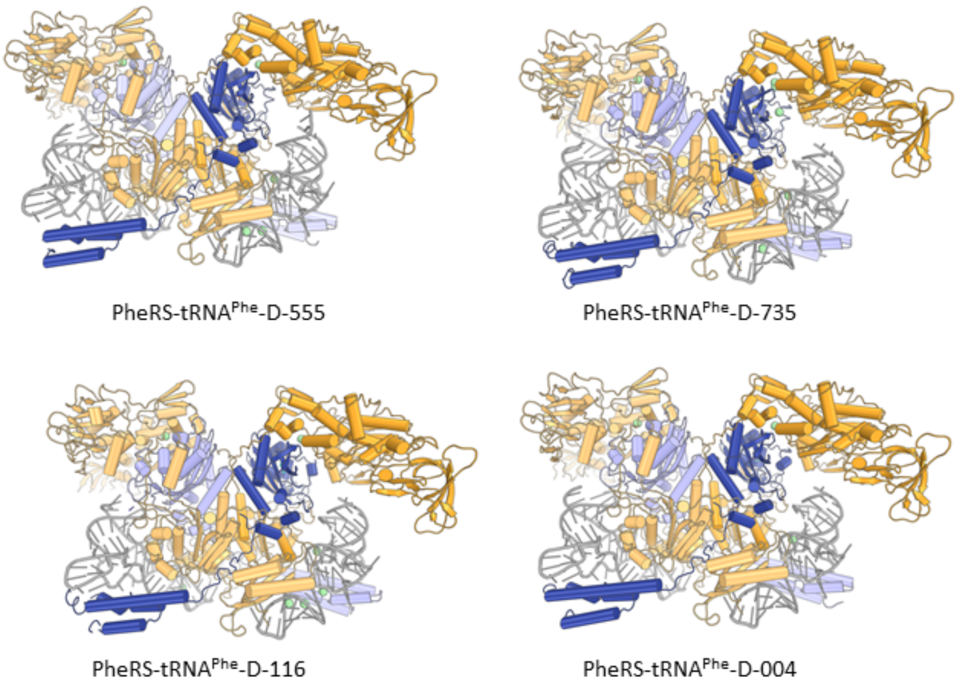

Figure S4. Overall structure of heterotetrametric *Mt*PheRS/tRNA^{Phe} complex bound to fragments D-555, D-735, D-116 and D-004 respectively. Blue, α-subunit; orange, β-subunit; gray, tRNA^{Phe}. Mg^{2+} ions are represented as light green spheres. The fragments are shown as sticks.

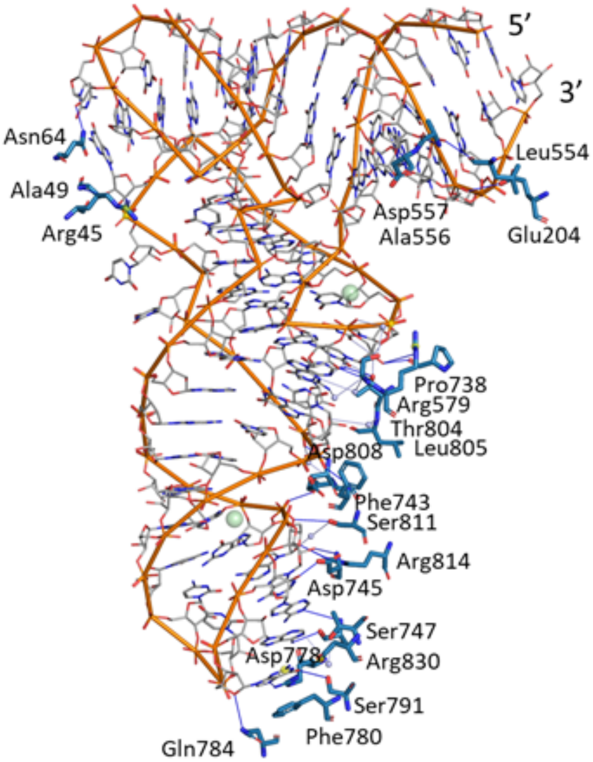

Figure S5. Residues of *Mt*PheRS (blue colored) interacting with $tRNA^{Phe}$. Hydrogen bond and water bridge interactions are shown as blue and violet lines respectively. Mg^{2+} ions are represented as light green spheres.

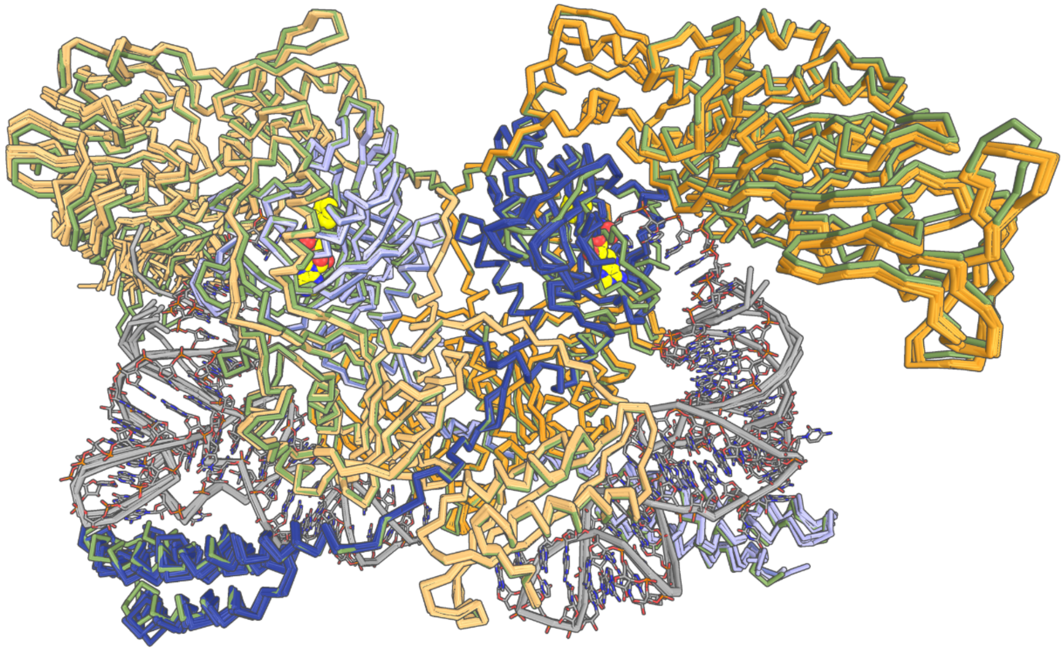

Figure S6. Structural overlay between the Phe-AMS (PDB ID: 7K98) and fragment bound *Mt*PheRS/tRNA^{Phe} complexes. Fragment bound structures are colored same as shown in Fig. S1. The *Mt*PheRS in Phe-AMS bound PheRS/tRNA^{Phe} structure is colored green.

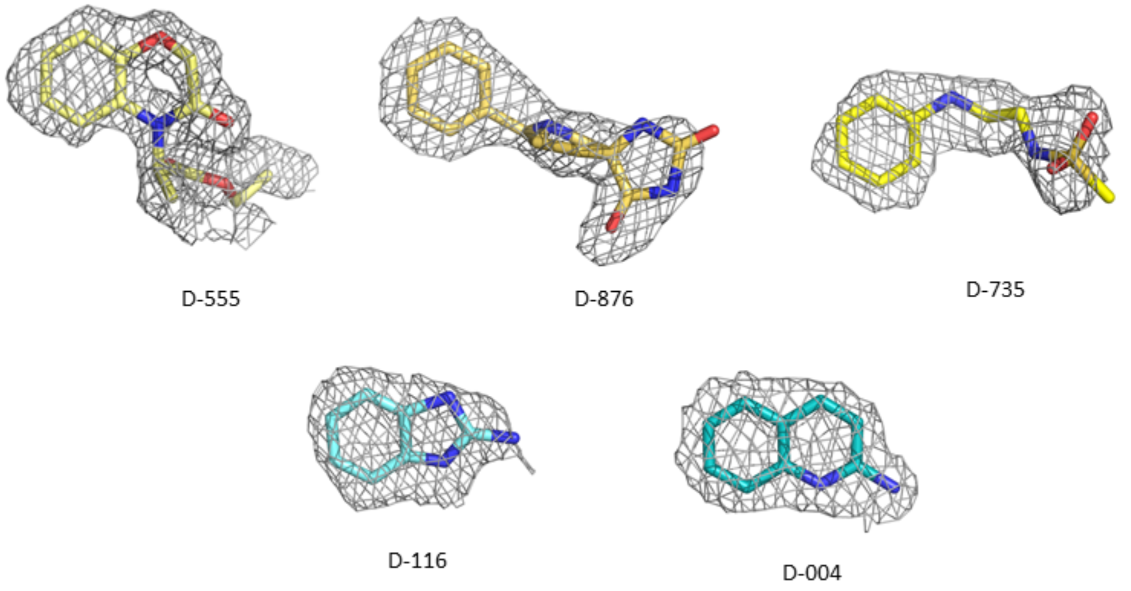

Figure S7. A $2F_o$-F_c electron density map contoured at 1.0 σ is shown in gray around the *Mt*PheRS binding fragments. The fragments are shown as stick model.

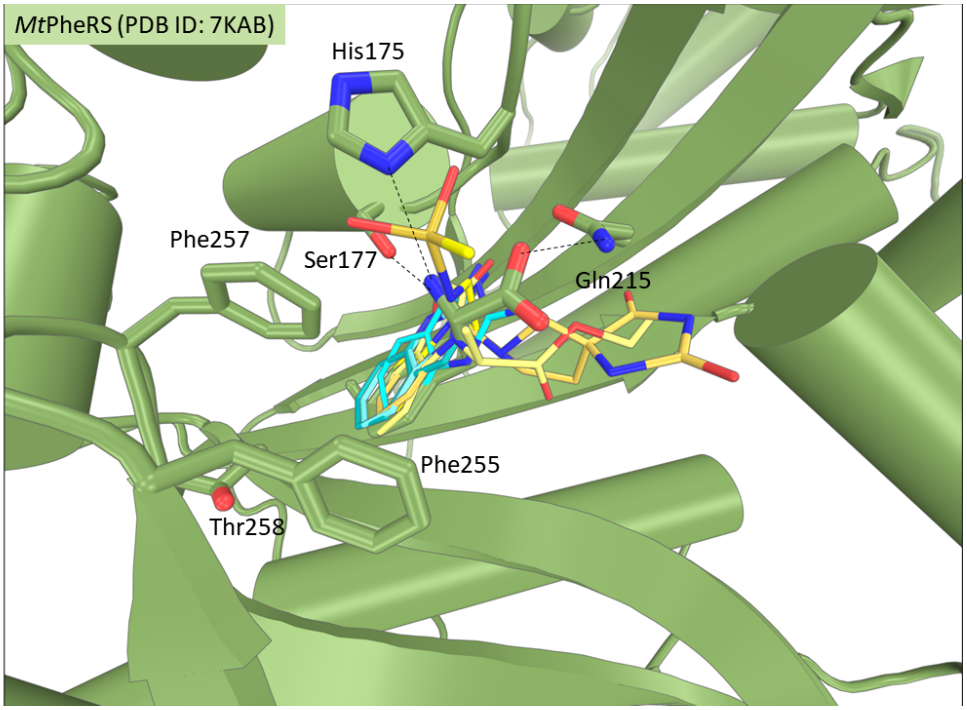

Figure S8. Close-up view of L-Phe and fragments binding to *Mt*PheRS/tRNA^{Phe} complex. L-Phe and interacting residues of *Mt*PheRS are shown as green sticks. Fragments determined by NMR and SPR are shown as yellow and cyan lines respectively.

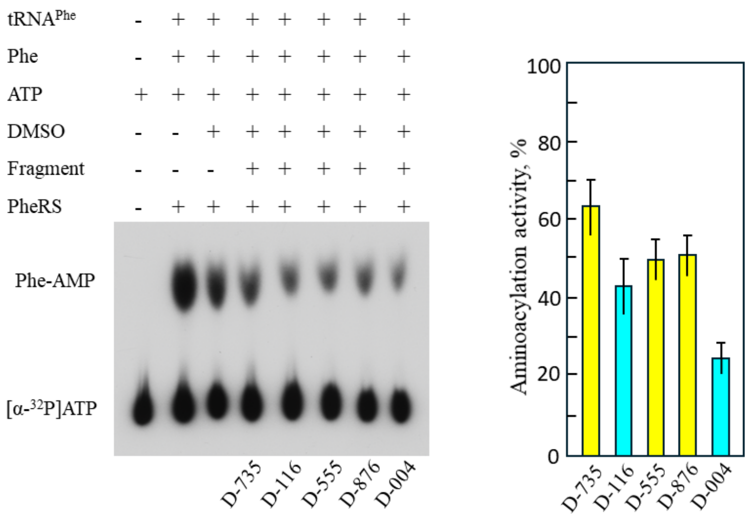

Figure S9. Aminoacylation inhibition by 4.5 mM fragments in the presence of 10% DMSO. *Mt*PheRS retained 64, 45, 50, 51, and 25% aminoacylation activity in presence of D-735, D-116, D-555, D-876, and D-004 fragments respectively

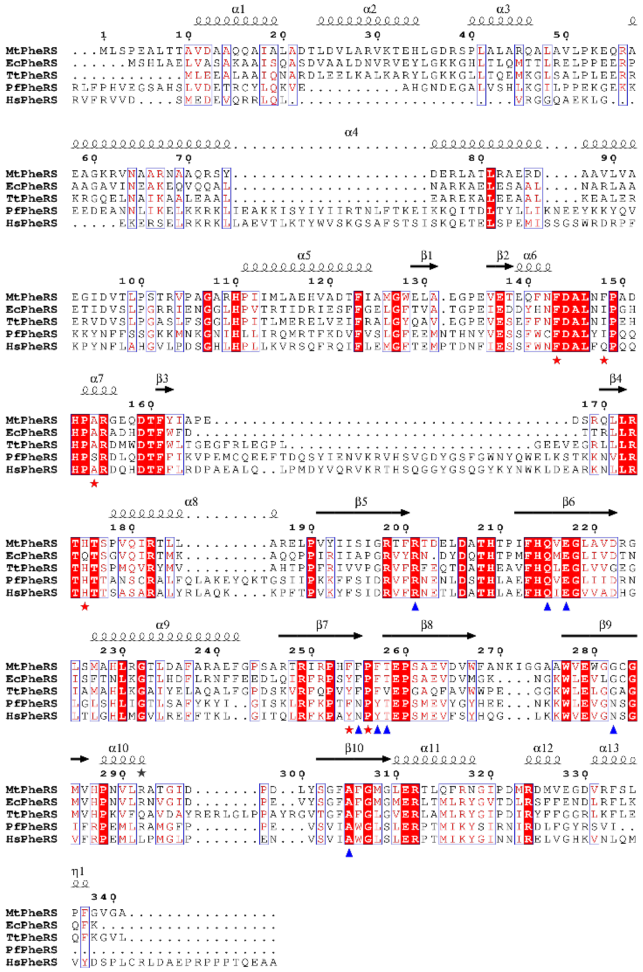

Figure S10. Structure based sequence alignment of PheRS alpha subunit from *M. tuberculosis* (*Mt*), *E. coli* (*Ec*), *T. thermophilus* (*Tt*), *P. horikoshii* (*pf*) and *H. sapiens* (*Hs*), with the secondary structure of *Mt*PheRS indicated above. The hydrophobic residues in the auxiliary pocket of *Mt*PheRS are highlighted as red asterisks, while the fragment interacting residues of *Mt*PheRS are highlighted as blue triangles.

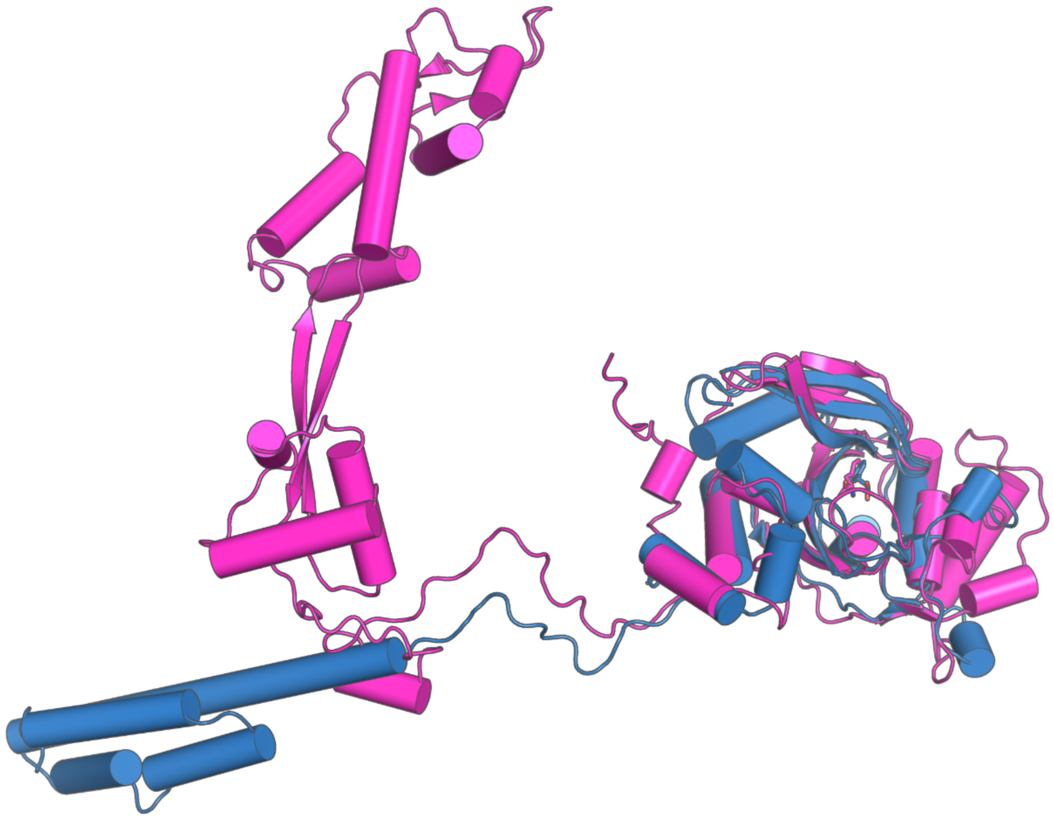

Figure S11. Superimposition of the alpha subunit structures from *MtPheRS* (blue; PDB ID: 7KAB) and *HsPheRS* (magenta; PDB ID: 3L4G). The L-Phe in the catalytic α2 domain is shown as sticks.

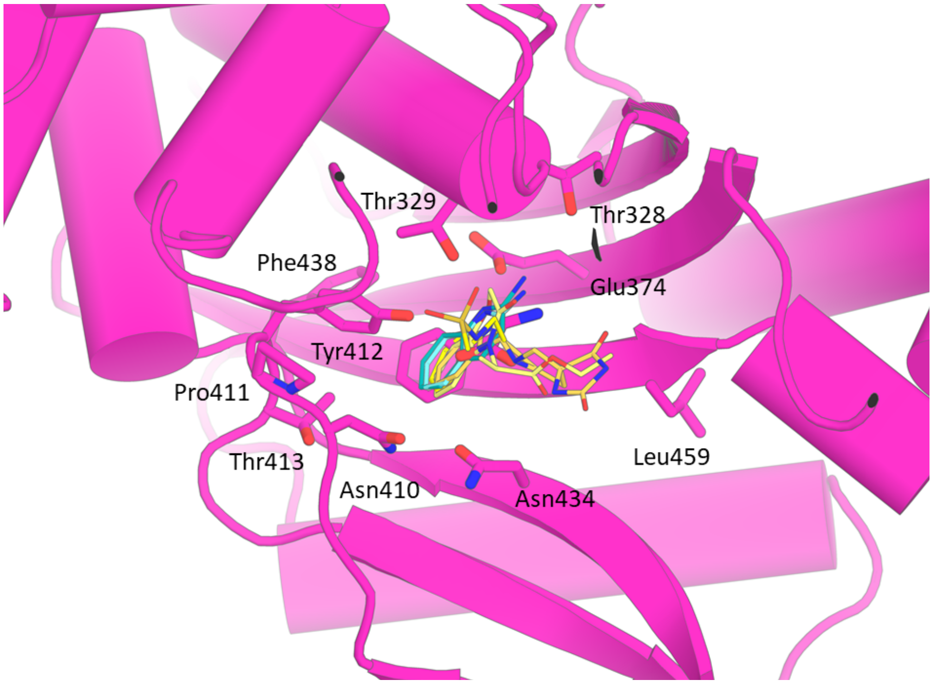

Figure S12. Superimposition of L-Phe bound *Hs*PheRS structure with fragment bound *Mt*PheRS/tRNA^{Phe} structures. Residues surrounding the L-Phe binding pocket of α2 domain of *Hs*PheRS are shown as magenta sticks. NMR and SPR determined fragments are shown as yellow and cyan lines respectively.

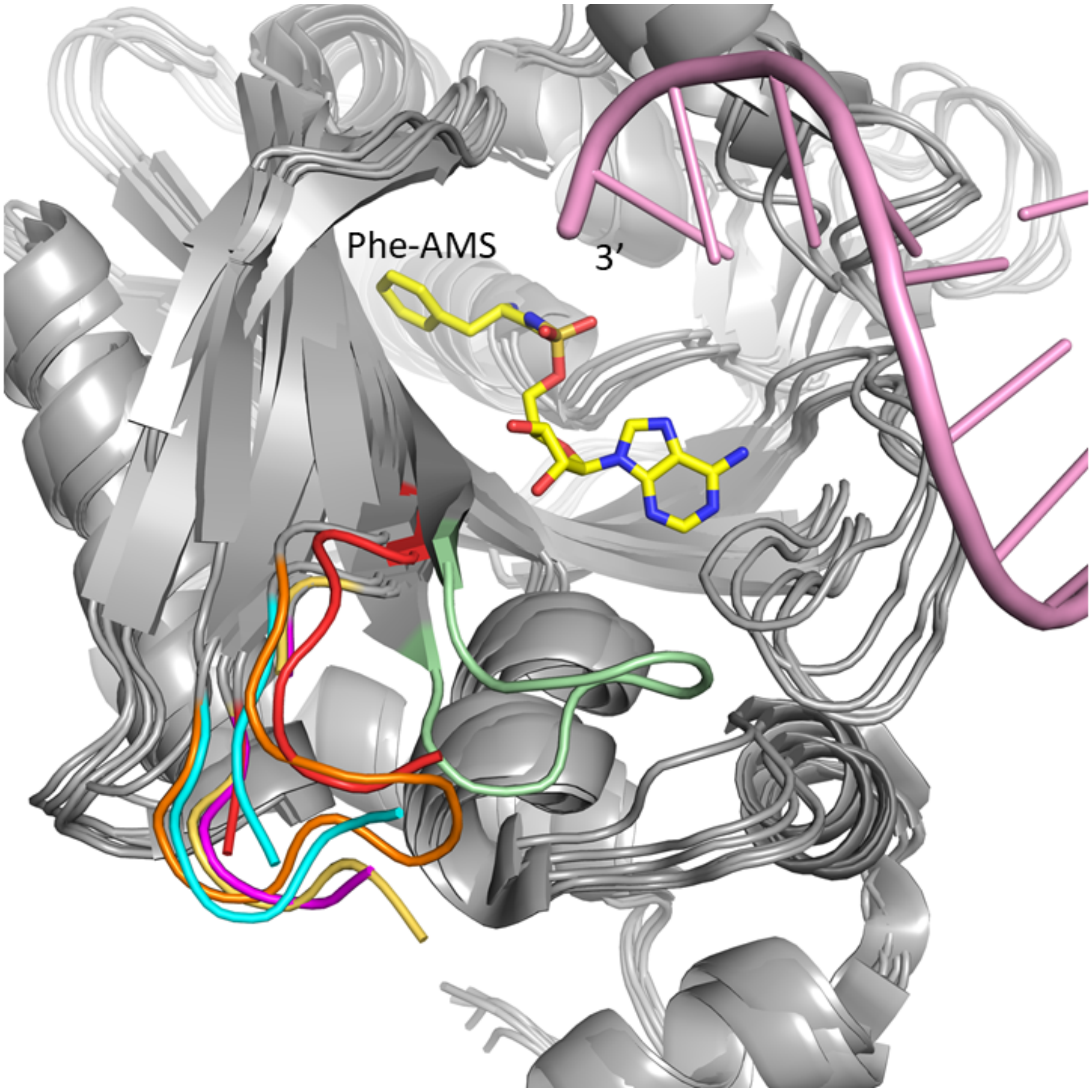

Figure S13. Structural comparison of the β-hairpin in the α2 domain of *Mt*PheRS/tRNA^{Phe} structures bound to Phe-AMS (light green; PDB ID 7K98), D-004 (orange), D-116 (red), D-555 (yellow), D-876 (magenta) and L-Phe (cyan; PDB ID: 7KA0). The Phe-AMS is shown as stick model. The tRNA^{Phe} binding the synthetic site in the Phe-AMS bound *Mt*PheRS/tRNA^{Phe} structure is shown in pink.

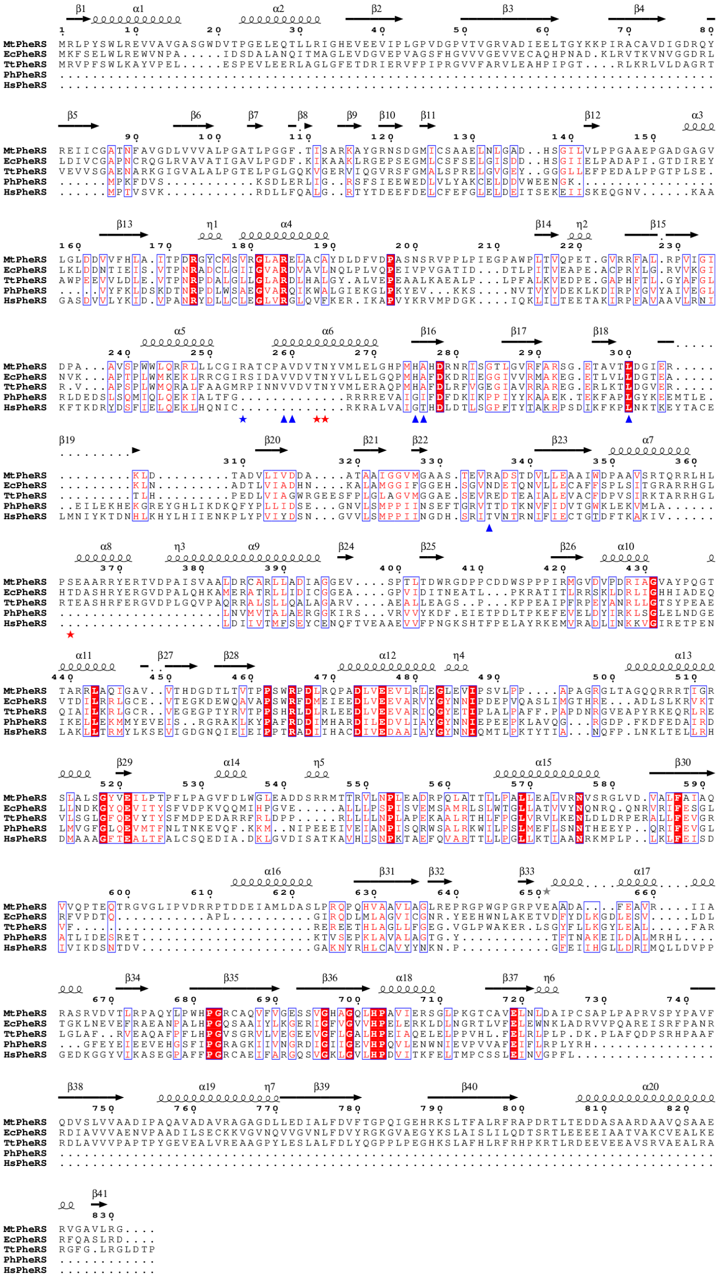

Figure S14. Structure based sequence alignment of PheRS beta subunit from *M. tuberculosis* (*Mt*), *E. coli* (*Ec*), *T. thermophilus* (*Tt*), *P. horikoshii* (*ph*) and *H. sapiens* (*Hs*), with the secondary structure of *Mt*PheRS indicated above. Blue triangles, residues at the β3/4 interface interacting with terminal A76; red asterisks, residues involved in positioning of catalytic water molecules at the editing site; blue asterisk, arginine interacting with C75 nucleotide of tRNA.

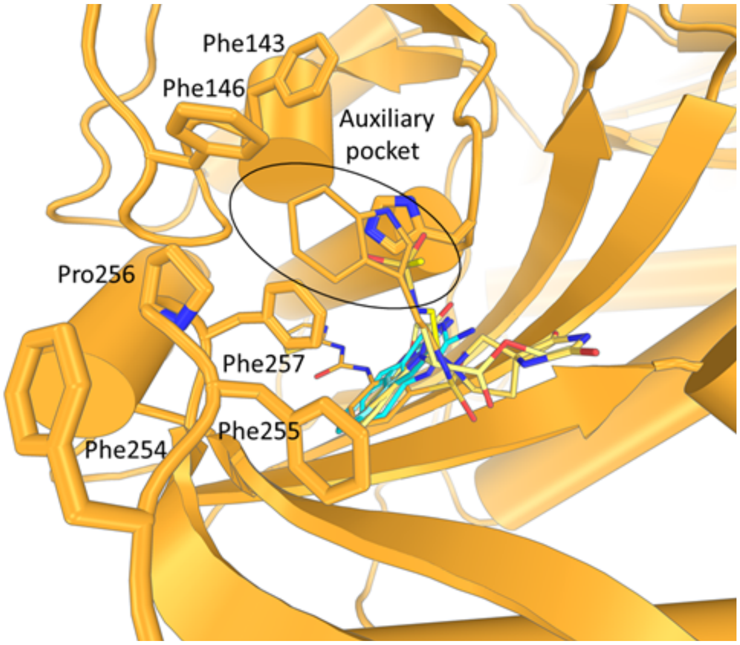

Figure S15. Close-up view of the binding of GDI05-001 compound in the catalytic site of α subunit of *Mt*PheRS. The structures of *Mt*PheRS/tRNA^{Phe} bound to fragments were superimposed with the GDI05-001 bound *Mt*PheRS (PDB ID: 7DB7). Both the GDI05-001 and fragments are shown as sticks. The fragments are shown as yellow (NMR determined) and cyan (SPR determined), while the GDI05-001 is shown in orange. The residues of *Mt*PheRS in the auxiliary pocket interacting with GDI05-001 is illustrated as sticks.

Table S1. List of fragments displaced by Phe-AMS in the NMR screening.

SMILES	Fragment ID	Selected for crystallization	Dose response in SPR
CN[C@]1(C2=CC=CC=C2)C[C@@H](O)C1	DDD01512419		
O=C(CC1=C(O)N=CC=C1)NC1=CC=CC=C1	DDD01512407		
O=C(NC1=CC=CC=C1)[C@@H]1C[C@H]2C[C@H]2N1	DDD01512399		
CN(C)C(=O)[C@H]1C[C@@H]1C1=CC=CC=C1	DDD01512377		
CNC(CO)C1=CC=CC=C1	DDD01305764		
NC(=O)C1COC2=CC=CC=C2O1	DDD01305754		
NC1CCCC1C1=CC=C(F)C=C1	DDD01305708		
O=S1(=O)CC(O)C(NCC2=CC=CN=C2)C1	DDD01305707		
CNC(C1=CC=NC=C1)C1=CC=C(Cl)C=C1	DDD01305679		
O=C1NC2=CC=C(Cl)C=C2C2(CCNCC2)O1	DDD01305661		
NC1=NC=C2C=CC=CC2=N1	DDD01305616		Y
O[C@H]1CCCC[C@H]1NCC1=CC=CC=C1	DDD01305608		
CN(C)C(=O)CC(N)C1=CC=CC=C1	DDD01305595		
C1=CC=C2NC(C3CNC3)=NC2=C1	DDD01305584		
NC[C@@H](O)C1=CC=CC=C1	DDD01305575	Y	
OC1=NC=NC2=C1CN(CC1=CC=CC=C1)CC2	DDD01305565		
CC(=O)N1CC2CNCCCC2(CO)C1	DDD01085540		
CC1NS(=O)(C2=CC=C(Cl)C=C2)=NC1=O	DDD01085501		
O=C1CNS(=O)(C2=CC=C(Cl)C=C2)=N1	DDD01085500		
CN1CCN(CC2=CC=CC=C2O)CC1	DDD01024716		
CC1=NC(C)=C(C(C)(O)C2=CC=CC=C2)S1	DDD01012604	Y	
O=S(=O)(C1=C(F)C=CC=C1)N1CCOCC1	DDD01012350	Y	
CS(=O)(=O)N1CCC(NC2=CC=CC=C2)C1	DDD01008882	Y	

O=C1NC(=O)C2(CCN(CC3=CC=CC=C3)C2)N1	DDD01008876	Y	
CCNC(=O)N1CCC(C2=CC=CC=C2)C1	DDD01008856	Y	
CNC(C1=CC=C(F)C=C1)C1=NC=CN=C1	DDD01008828	Y	
NC1=CC=CC2=NC=CN12	DDD01008820		Y
CN1N=CC2=CC=C(C(=O)N3CCCC3)C=C21	DDD01008800		
N#CC1(C2=CC=CC=C2)CCNCC1	DDD01008781		Y
CN(C)C(=O)C1=CNC2=C1C=CC=C2	DDD00933655		
O=C1NC2=C(C=CC=C2)CO1	DDD00920317		
OC(C1=CC=CC=C1)C1=CC=CN=C1	DDD00910107		
NC1=CN=C2C=CC=CC2=N1	DDD00813183		
NC(=O)C(C1=CC=CC=C1)C1=NC=CC=C1	DDD00808251		
CNC(=O)C1CC(=O)N(CC2=CC=CC=C2)C1	DDD00805774	Y	
CS(=O)(=O)N1CCN(CC2=CC=CC=C2)CC1	DDD00805755	Y	
CS(=O)(=O)NCCNC1=CC=CC=C1	DDD00805735	Y	
CS(=O)(=O)NCC(O)C1=CC=CC=C1	DDD00805734	Y	
CS(=O)(=O)NCCC1=CC(O)=CC=C1	DDD00805733		
O=C1N[C@@H](C2=CC=CC=C2)CO1	DDD00771548		
OC1=NC=NC2=C1C=CS2	DDD00771521		
CS(=O)(=O)C1=CC=C(O)C=C1	DDD00771463		
NC1=CC=CC2=CC=CN=C12	DDD00771435	Y	
CN(C)C(=O)C1=NN(C)C2=CC=CC=C21	DDD00771060	Y	
O=C(NC1=CC=CC=C1)C1=CC=NO1	DDD00749416	Y	
NC1=NN(CC2=CC=C(F)C=C2)C=C1	DDD00321519		
OC1=CC=CC=C1N1CCOCC1	DDD00175967	Y	
OC1=CC=NC2=CC=CC=C12	DDD00122023		
CS(=O)(=O)NCC1=NC(C2=CC=CC=C2)=NO1	DDD00108567		
CCOC(=O)C(C)N1C(=O)COC2=C1C=CC=C2	DDD00107555	Y	
CC1=NC(CN2C(=O)COC3=C2C=CC=C3)=CC=C1	DDD00107499		

O=C1COC2=C(C=CC=C2)N1CC1=CC=CC=N1	DDD00107367		
CN1N=CC2=C1CCCCN2CC1=CC=CC=C1	DDD00107353		
O=C1COC(CC2=CC=CC=C2)CN1	DDD00107283		
CC1=CC(CN2C(=O)COC3=C2C=CC=C3)=NO1	DDD00107199	Y	
CC(C)NC(=O)N1C(=O)OC2=CC=CC=C21	DDD00102232		
CCNC(=O)N1C(=O)OC2=CC=CC=C21	DDD00102226		
CNC(=O)N1C(=O)OC2=CC=CC=C21	DDD00102225		
CN1N=NC(C2=CC=C(O)C=C2)=N1	DDD00101091		
O=C1NC2=CC=CC=C2O1	DDD00100750		
OC1CCCN(CC2=CC=CC=C2)C1	DDD00100736	Y	
NCC1=CC=CC=C1COC1=CC=CC=C1	DDD00100728		
O=S(=O)(C1=CC(F)=CC=C1)N1CCCC1	DDD00100722		
OCC1CCN(CC2=CC=CC=C2)CC1	DDD00100707		
CNCC1=CC=CC(C2=CN=CC=C2)=C1	DDD00100601		Y
CNCC1=CC=CC(C2=CC=NC=C2)=C1	DDD00100593		Y
O=C1CN(C(=O)OCC2=CC=CC=C2)CCN1	DDD00100439		
N#CC1=CC=C(OC2=CC=CC=N2)C=C1	DDD00100415		
CNCC1=CC=CC(C2=CSC(C)=N2)=C1	DDD00100406		Y
NC1=CC=C2N=CSC2=C1	DDD00100392	Y	
NC1=CC=C2N=CC=NC2=C1	DDD00100369		
OCC1=C(N2C=CC=C2)C=CS1	DDD00100351		
O=C(O)C1=NN2C(=C1)N=CC=C2C1=CC=CC=C1	DDD00100341	Y	
CCN1CCN(C(=O)C2=CC=C3NC=CC3=C2)CC1	DDD00100334		
FC1=CC=C(CNC2=NC=CC=N2)C=C1	DDD00100305		
CC1CC(C2=CC=CC=C2)N2N=CN=C2N1	DDD00100289		
CC1=NC2=NC=NN2C(N)=C1	DDD00100287		
OC1=NC=NC2=CC=CC=C21	DDD00089329		

C1=CC=C(NCC2=CC=CN=C2)C=C1	DDD00086019		
OCC1=NC=CN1CC1=CC=CC=C1	DDD00086014		
O=C1OC2=CC=CN=C2N1CC1=CC=CC=C1	DDD00084182	Y	
NC1=NC2=C(C=CC=C2)C=C1	DDD00079004	Y	Y
CN(C)CCOC1=C2C(=CC=C1)N=C(N)N=C2N	DDD00072751		
NC1=NC2=C(C=CC=C2)N1CC1=CC=CC=N1	DDD00070359		
C1CCC2C(C1)NC([nH]2)N	DDD00067116	Y	Y
OC1=C2C(F)=CC=CC2=NC=N1	DDD00060034		
CCOC(=O)CN1C(=O)COC2=C1C=CC=C2	DDD00024969	Y	
CC1=NC2=C(C=CC=C2)C(N2CCNCC2)=C1	DDD00005864		
CC1(C)NC2=CC=CC=C2NC1=O	DDD00003339		

Table S2. List of unmodelled residues

	Chain	Residue range
PheRS/tRNA^{Phe}-D-555	A	48-61
	D	273-275
	E	57-68
	E	111-123
	E	136-137
PheRS/tRNA^{Phe}-D-735	D	272-275
	E	61-67
	E	85-87
	E	114-120
	E	136-137
PheRS/tRNA^{Phe}-D-876	A	55-61
	D	272-275
	E	57-68
	E	84-87
	E	111-121
	E	136-137
PheRS/tRNA^{Phe}-D-116	A	5-7
	A	269-273
	E	61-68
	E	76-77
	E	85-87
	E	112-121
	E	135-139
PheRS/tRNA^{Phe}-D-004	E	61-69
	E	112-121
