# Supplementary material for: Different chemical scaffolds bind to L-phe site in Mycobacterium tuberculosis Phe-tRNA synthetase
Source: Eur J Med Chem. Author manuscript; Available in PMC 2025 Sep 19. (PMC12447630; doi:10.1016/j.ejmech.2025.117335)
Supplement: PheRS_FraScr_Fig.S16 [file NIHMS2096802-supplement-PheRS_FraScr_Fig_S16.docx]

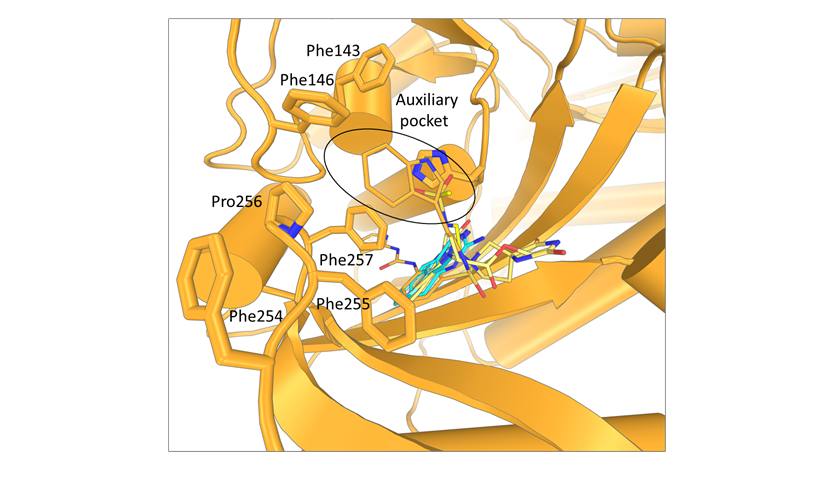


**Figure S16.** Close-up view of the binding of GDI05-001 compound in the catalytic site of α subunit of *Mt*PheRS. The structures of *Mt*PheRS/tRNA^Phe^ bound to fragments were superimposed with the GDI05-001 bound *Mt*PheRS (PDB ID: 7DB7). Both the GDI05-001 and fragments are shown as sticks. The fragments are shown as yellow (NMR determined) and cyan (SPR determined), while the GDI05-001 is shown in orange. The residues of *Mt*PheRS in the auxiliary pocket interacting with GDI05-001 is illustrated as sticks.
